# Rapid Assessment of Carotenoid and Gluten Quality Traits in Durum Wheat Hybrids Confirmed by Biochemical and Molecular Marker Analyses

**DOI:** 10.3390/life16101700

**Published:** 2026-10-10

**Authors:** Maral Utebayev, Svetlana Dashkevich, Oksana Kradetskaya, Irina Chilimova, Ruslan Zhylkybaev, Tatyana Zhigula, Tatyana Shelayeva, Gulmira Khassanova, Marzhan Kuzbakova, Botagoz Sharipova, Yuri Shavrukov

**Affiliations:** 1 A.I. Barayev Research and Production Centre of Grain Farming, Shortandy 021601, Kazakhstan; vetka-da@mail.ru (S.D.); oksana_cwr@mail.ru (O.K.); coronela@mail.ru (I.C.); gaziz-amonik@mail.ru (R.Z.); tanya.zhigula@mail.ru (T.Z.); tatyana.shelaewa@yandex.kz (T.S.); 2 Institute of Agronomy and Forestry, S. Seifullin Kazakh AgroTechnical Research University, Astana 010000, Kazakhstan; g.hasanova@kazatu.edu.kz (G.K.); m.kuzbakova@kazatu.edu.kz (M.K.); 3 Biology Department, Sh. Ualikhanov University, Kokshetau 020000, Kazakhstan; oralovna8282@gmail.com; 4 College of Science and Engineering, Biological Sciences, Flinders University, Adelaide, SA 5042, Australia

**Keywords:** carotenoids, colorimetric method, durum wheat, genotyping, gluten, Glutomatic-2200 system, seed quality, SDS sedimentation

## Abstract

High levels of carotenoids and gluten are the most important traits for seed quality and colour of semolina produced from durum wheat (*Triticum durum* Desf.). Rapid methods based on colourimetry with three coordinates L*a*b* and SDS sedimentation produce indirect measurements of carotenoids and gluten, respectively. In contrast, traditional and accurate biochemical methods with *n*-butanol extraction of carotenoids and a Glutomatic-2200 instrument system for gluten are more complicated approaches requiring specific equipment and much more time. The aim of this study was to test and compare rapid and traditional methods for carotenoid and gluten quality in 8 durum wheat cultivars used as parents and 10 F_3_-F_4_ hybrids, which were very diverse in their composition. Genotyping of the *TdPsy-B1* and *TdGlu-B1* genes using SNP-based ASQ markers was consistent with carotenoid content and gluten quality in the parental lines and hybrids. Strong associations were found in the comparison between rapid measurements and traditional methods for carotenoid and gluten quality. Therefore, colourimetric and SDS sedimentation methods can be used for approximate assessment of carotenoid and gluten quality in durum wheat, but their practical value lies primarily in the ability to rapidly rank and select genotypes for subsequent detailed analysis.

## 1. Introduction

Among cereal crops, wheat is one of the most important foods for 1/3 of the world’s population, second only to rice. Globally, hexaploid bread wheat (*Triticum aestivum* L.) accounts for approximately 95% of total wheat production. Therefore, the remaining 5% of wheat production is related to tetraploid durum wheat *(Triticum durum* Desf.), with 2n = 4x = 28 chromosomes and an AABB genome, and is the only tetraploid wheat species used primarily for pasta production [1]. However, depending on the cultivation region, grains are also suitable for bread baking and the production of groats or hulled grains. The main producers of durum wheat are countries in Europe (Italy, Spain, Portugal and France), North Africa (Algeria, Morocco, Libya and Tunisia), and the Middle East (Türkiye and Syria), as well as Australia [2], Kazakhstan and Russia [3]. A combination of high grain hardness, vitreousness, and elevated levels of carotenoids and protein in the grain of durum wheat is very suitable for production of semolina, which is the main raw material for manufacturing high-quality pasta products including spaghetti and penne. The nutritional value of durum wheat grain is based on 12–16% protein, about 70% carbohydrates, 1.9% fat, 1.6% dietary fibre, and 1.6% minerals [4]. In addition to macronutrients, grains of durum wheat are rich in provitamin A, antioxidants, and anthocyanins, which help protect cells from oxidative stress. Compared to other wheat-based products, pasta has a longer shelf life. Pasta and other products of durum wheat are among the most popular food products, with superior taste, texture and rheological properties, as well as the potential to incorporate functional ingredients [1].

Carotenoid pigments are responsible for the yellow colour of seeds and their processed products, and this trait has high importance for consumers and processing properties among biochemical components of durum wheat seeds [5]. Carotenoids are lipophilic natural pigments belonging to the class of isoprenoids (terpenoids), and they are tetraterpenoids with a C_40_ carbon skeleton formed by eight isoprene units. Based on their chemical structure, carotenoids are classified into two main groups: hydrocarbon carotenes and oxygen-containing xanthophylls [6]. In durum wheat, carotenoids include β-carotene, zeaxanthin, β-cryptoxanthin, β-apocarotenal, antheraxanthin, taraxanthin (lutein-5,6-epoxide), flavoxanthin, and triticoxanthin, but the predominant component is lutein [7].

The content of carotenoid pigments in durum wheat grain is a quantitative trait characterised by significant genotypic variability, while environmental conditions can make a substantial contribution to its phenotypes [8,9]. For example, the carotenoid content can range from 2.2 to 14.2 ppm, depending on the genotype and the environment [10,11,12].

Undoubtedly, chromatographic measurement of the carotenoid profile makes it possible to determine the composition qualitatively and quantitatively, identify minor compounds, and characterise genotypes of durum wheat [10]. However, despite the high quality of analytical data resulting from chromatographic methods, their application to the screening of multiple durum wheat breeding lines is limited by the high cost of analysis, the complex sample preparation, and significant time requirements.

The most common method for the measurement of carotenoid content remains the ICC method, Standard 152 [13]. The method is based on extraction of the total carotenoids from durum wheat grain using water-saturated *n*-butanol, followed by the determination of optical density in the 435.8–440 nm spectral range using a spectrophotometer together with AACC-approved methods of analysis (AACC Colour and pigments methods, standard 14-50.01) [14]. To work with the limited grains available, this method was modified using 2–4 g, which significantly lowered solvent consumption and increased throughput [15].

Another method for indirectly assessing breeding material for the presence of yellow pigment (carotenoids) is colourimetric. This method is based on the evaluation of colour characteristics of the whole-crushed grains and milled products (semolina) with the CIE L*a*b* colour coordinates using a Minolta chromameter or other portable colourimeter. The colourimetric measurement coordinate values for L* (lightness: from 0 for black to 100 for white), a* (red–green axis), and b* (yellow–blue axis) are in the Munsell colour system [16]. For durum wheat, the b* coordinate is the most informative, characterising the degree of yellow colour for the sample. The increase in the b* value corresponds to higher yellow colour intensity of the semolina [11].

Alongside carotenoids, endosperm storage proteins make a significant contribution to the processing quality of durum wheat. Glutenins are of particular importance, as they influence the structural and mechanical properties of gluten and, consequently, the quality of semolina and the final durum wheat products [9]. A combination of physicochemical and rheological methods is used to assess the quality of durum wheat gluten. One of the most common indicators is the sedimentation test value with sodium dodecyl sulfate (SDS). The method is based on the ability of gluten proteins to absorb water in the presence of lactic acid and SDS. Stronger gluten exhibits a greater capacity for absorption and forming a stable protein complex, and consequently, the particles settle-down more slowly and form a larger volume of sediment. In accordance with the ICC 151 standard, a higher SDS value corresponds to stronger gluten strength, and it is used as an indicator of protein quality in durum wheat [17]. This classic method was originally developed to assess the baking quality of soft wheat [18] and it subsequently gained popularity in breeding programs [19,20]. This method was further used to evaluate glutens in durum wheat and gained widespread adoption for its suitability for small sample sizes, the simplicity of the analysis, and the relatively high throughput [21,22,23,24]. It is important to note that SDS sedimentation is an indirect indicator of gluten strength and does not reflect the full range of its viscoelastic properties. Therefore, for a detailed quality assessment, it is advisable to supplement it with rheological methods. At the same time, the simplicity, reproducibility, and ability to analyse small sample sizes make SDS-sedimentation an effective screening method for assessing gluten strength, especially when dealing with large numbers of breeding samples.

Glutenins are polymeric protein fractions of gluten, consisting of high-molecular-weight (HMW) and low-molecular-weight (LMW) glutenin subunits (GS). In tetraploid wheat, HMW-GS are encoded by the *Glu-A1* and *Glu-B1* loci, whereas LMW-GS are primarily associated with the *Glu-A3* and *Glu-B3* loci [25]. The allelic polymorphism of these loci determines the variability in the subunit composition of high- and low-molecular-weight glutenins and may be associated with differences in gluten strength and dough rheological properties. When evaluating durum wheat accessions based on the gluten index (GI), the following allele combination ranking was established for the low-molecular-weight (LMW) glutenin alleles influencing this trait: *Glu-A3c*, *Glu-B3a*, *Glu-B2a* = *Glu-A3a*, *Glu-B3a*, *Glu-B2a* > *Glu-A3b*, *Glu-B3b*, *Glu-B2a* and *Glu-A3a*, *Glu-B3a*, *Glu-B2a* > *Glu-A3c*, *Glu-B3f*, *Glu-B2a*. For high-molecular-weight (HMW) glutenin, the following relationship between subunits was observed: 6 + 8 (*Glu-B1d*) = 7 + 8 (*Glu-B1b*) > 20 + 20 (*Glu-B1e*) [26,27]. Thus, the combined effect of various glutenin subunits must be considered to form a comprehensive assessment of gluten quality. While the traditional identification of alleles using SDS-PAGE is very informative, the simultaneous use of different allele-specific markers can be helpful for selection of promising genotypes.

The developed DNA markers Glu-Ax1/2*_SNP and BX7OE_866_SNP demonstrated a significant positive association between *Glu-A1a* allele and the gluten index GI, as well as a negative effect of *Glu-A1c* on GI. Additionally, *Glu-B1al* and *Glu-B1f* alleles showed a beneficial effect on protein and gluten content. Notably, genotypes carrying the *Glu-A1c* + *Glu-B1al* allele combination were shown to fall into the high-quality group [28]. GWAS identified numerous SNP associations with grain and semolina quality traits. Regarding gluten strength, the most significant associations were localised on chromosomes 1AS and 1BS, where 95 and 84 significant SNPs, respectively, were detected in regions containing *Glu-3/Gli-1* gene clusters. Genomic prediction resulted in accuracies of 0.66 for sedimentation volume, 0.44 for protein content, 0.45 for bulk density, 0.53 for semolina yield, and 0.51 for semolina colour [29].

The molecular-genetic approach is equally important for studying the carotenoid trait. The genetic control of carotenoids in durum wheat grain is complex and involves several loci. One of the key genes, *Psy1*, encodes phytoene synthase, an enzyme involved in the early stage of carotenoid biosynthesis. A marker has been developed for the *Psy1-A1o* allele, which is associated with higher carotenoids content [30]. It should be noted that the genetic control of carotenoid accumulation is mediated by genes located on different chromosomes [5].

Recent studies have demonstrated the importance of using molecular markers to improve grain colour and gluten quality in durum wheat [8,9,15]. Researchers have focused primarily on investigation of genetic diversity, validation of molecular markers, and identification of associations between specific loci and quality parameters [28,29,30]. At the same time, the application of comprehensive screening, combining the rapid assessment of phenotypic traits with biochemical confirmation and allele-specific genotyping in durum wheat breeding material, remains insufficiently studied. Such an approach is particularly promising during early stages of plant selection, when it is necessary to evaluate large amounts of segregating genetic material with a limited quantity of seeds available for analysis.

To address this issue, in the present study, 10 F_3_-F_4_ durum wheat hybrid populations and their parents were evaluated using a comprehensive approach that included rapid grain colour assessment via the CIELAB method, SDS sedimentation, traditional biochemical methods to determine carotenoid content and gluten quality parameters, and genotyping of studied accessions using ASQ markers for *TdPsy-B1* and *TdGlu-B1* genes.

The aim of the study was a comprehensive evaluation of breeding material at the early stages of selection using rapid screening for carotenoid content and gluten quality. The following objectives included: (1) to determine the relationship between CIELAB colour characteristics and carotenoid content measured using the standard water-saturated *n*-butanol extraction method; (2) to evaluate the relationship between the SDS-sedimentation value and gluten quality determined using the Glutomatic-2200 system; and (3) to investigate the association of these phenotypes and the allelic diversity of *TdPsy-B1* and *TdGlu-B1* genes using ASQ-type SNP markers in parents and F_3_-F_4_ hybrid populations.

## 2. Materials and Methods

### 2.1. Plant Material and Hybrid Production

Seeds of eight durum wheat cultivars (*Triticum durum* Desf.) used in this study were obtained from the Durum Wheat Breeding Laboratory of the A.I. Barayev Research and Production Centre of Grain Farming. The list and full description of the durum wheat accessions and hybrids used are present in Table 1.

In 2020, the plants were grown in the research field of the A.I. Barayev Research and Production Centre of Grain Farming (BRPCGF), Akmola Region, Kazakhstan (51°40′25.3″ N, 71°0′46.8″ E). Spikes were manually emasculated, covered by paper bags, pollinated under careful control to ensure no anthers remained in emasculated florets and finally covered by paper bags again until maturity. Ten crosses were successful (Table 1), and an average of 8–10 F_1_ hybrid seeds per cross were harvested. The hybridity of the obtained seeds was confirmed by gliadin electrophoresis in an aluminium lactate buffer (pH 3.1) according to [31]. F_1_ plants were then grown in greenhouse conditions to full maturity in pots containing soil collected from the same experimental field. Harvested F_2_ seeds were carefully bulked in each hybrid combination and sown in the next season in the same research field.

In 2024 and 2025, bulked F_3_ and F_4_ seeds in each hybrid population, respectively, were manually sown in the same research field, in two fully randomised plots with 20 rows, 1 m in length, 10 cm between rows, with 2 m^2^ area in each plot and a distance between plots of 50 cm. In each hybrid combination, both parents were grown close to their hybrids in the same field and for each parent. In each year, four seed bulks, two from each plot, were prepared for all hybrids and parents, and they were used for further analyses as eight biological replicates.

### 2.2. Weather Conditions of the Growing Seasons

In 2024–2025 seasons, weather conditions during the vegetative period of the studied accessions were variable in both temperature and moisture levels. According to regional agrometeorological data, the long-term average precipitation during the wheat growing season (May–August) was approximately 180–200 mm, with average air temperature 16.5–17.5 °C. In 2024, high moisture levels were recorded for the growing season, with total precipitation in May–August exceeding 309.1 mm, significantly higher than the long-term average and with an 18.2 °C average temperature. Such wet conditions were favourable for plant growth and grain filling. In 2025, the growing conditions were drier, with less precipitation for the corresponding period, with 79.9 mm and an average air temperature of 19.2 °C. The hydrothermal coefficient reached 0.33, indicating for moderate drought during the growing season for durum wheat plants.

### 2.3. Rapid Colourimetric and Traditional Biochemical Methods of Carotenoid Determination in Seeds

Samples of whole-ground grain from bulked plants were obtained using a Perten 3100 laboratory mill (Perten Instruments, Springfield, IL, USA) equipped with a 0.8 mm sieve. The rapid colourimetric method of whole-ground seeds from individual plants was used for yellow pigment content (YPC), using a Minolta chromameter CR-300 (Konica Minolta, Sydney, Australia).

**Figure 1:**
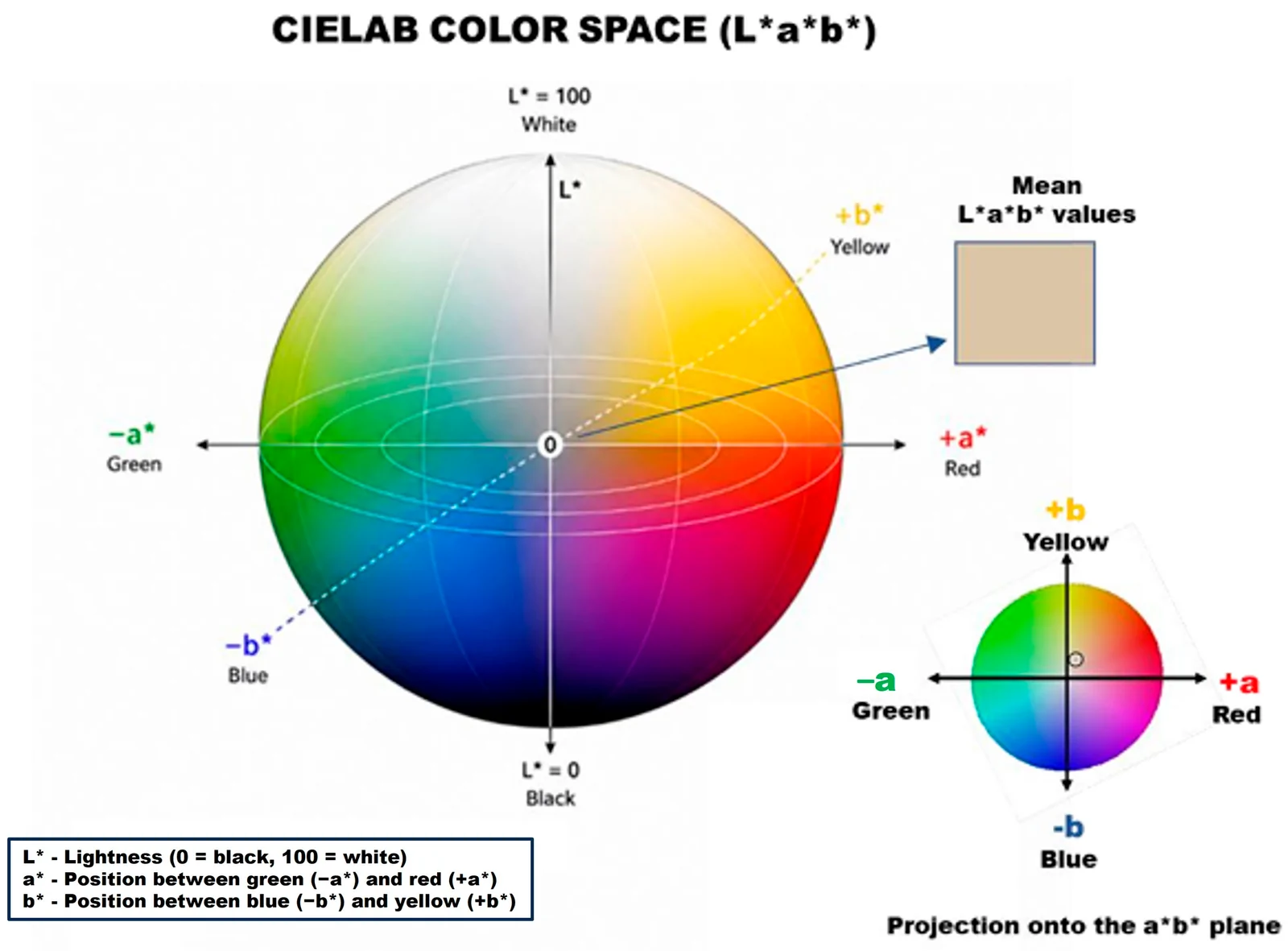
Visualisation of the CIELAB (L*a*b*) 3D colour coordinates with approximate location of whole-ground grain colour. The visual model was prepared using Artificial Intelligence program ChatGPT 4.

The colourimetric results were identified and automatically linked to the CIELAB system, showing each measured colour in three coordinates, as follows: (1) Brightness ‘L*’: from 100 white to 0 black; (2) Red-Green ‘a*’: from +60 red to −60 green; and (3) Yellow-blue ‘b*’: from +60 yellow to −60 blue. Before colourimetric measurement of samples, the Minolta instrument was calibrated once using the manufacturer-supplied white calibration plate with certified reference values under the CIE D65 standard illumination (L* = 83.4, a* = 0.3191, and b* = 0.3362), according to the manufacturer’s instructions. For each studied genotype, seeds were harvested from eight bulks described above, ground separately for colourimetric measurement, and each colour-coordinate result represented the average of three measurements (technical runs). The averaged L*a*b* colour coordinates were converted to the RGB (Red, Green, and Blue) colour model solely for visualisation purposes using the online service [32], and the resulting visualisations are present in Figure 1. The original L*a*b* values were used for the subsequent analyses. The mean L*a*b* colour-coordinate values measured in ground grain across all studied hybrid lines were L* = 79.53, a* = 0.76, and b* = 18.24 and were used as reference values for the comparison.

In addition to the colourimetric method, yellow pigments were extracted from single plant whole-ground grain samples using water-saturated *n*-butanol, WSB, according to procedures outlined in ICC standard 152 and AACC standard 14-50.01 [13,14] with minor modifications for the small number of seeds in each studied sample. From ground seeds, 1.0 g was used as a sample and placed into a 10 mL plastic tube with 5.0 mL of WSB [14]. The mixture was homogenised on a Multi RS-60 rotator (Biosan, Riga, Latvia) for 30 min, and then incubated in the dark for 16–18 h at +5 °C. The mixture was centrifuged at 3000 rpm for 20 min using an ELMI CM-6M centrifuge (Elmi, Riga, Latvia), and the supernatant was collected.

The carotenoid content was determined using a spectrophotometer (SF-2000, Spectr, St-Petersburg, Russia) with a supplied KU-10.10A quartz cuvette with 10 mm optical path length, and the extract optical density was measured at 436 nm. Supernatant absorbance was converted to yellow pigment content as specified in AACC 14-50.01 [14].

### 2.4. Rapid SDS-Sedimentation Test for Quality of Gluten

Gluten quality was assessed using the SDS-sedimentation test, based on wholemeal grain sample sedimentation in lactic acid [23], with some modifications. Ground grain sample (1.0 g) was placed in a 25 mL plastic tube with 4 mL of sterile water. The tube was vigorously shaken for 4 s and then incubated in an upright position at room temperature (22 °C) for 7.5 min followed by a repeat of shaking and incubation.

After the initial settling period, 12 mL of freshly prepared working solution was added. The working solution was prepared by mixing a 9.4% lactic acid solution with a 2% sodium dodecyl sulfate (SDS) solution at a volume ratio of 1:48. The tubes were mixed for 2 min and incubated as described above over 15 min for sedimentation. The height of the sediment was measured in millimetres. The obtained SDS-sedimentation value was converted into the equivalent of standard SDS-sedimentation values (mL) using the equation: *y* = 12.0 + 0.63*x*, where *x* is the sediment height in the used SDS sedimentation test (mm) and *y* is the converted SDS-sedimentation value (mL) [24].

### 2.5. Traditional Biochemical Analysis of Total Proteins and Glutens in Seeds

Protein content (PC) was determined by the Kjeldahl method using an automatic distillation unit UDK 142 (Velp Scientifica, Usmate Velate, Italy). A 0.03 g portion of the ground seed sample was transferred to a thermo-stable glass tube and subjected to acid digestion in the presence of 700 µL of concentrated sulfuric acid and 0.3 g of pre-dosed catalyst ground Kjetabs VCM tablets containing 3.5 g K_2_SO_4_ and 0.1 g CuSO_4_·5H_2_O. The mineralisation process comprised the following stages: Room temperature, 15–18 h; 100 °C, 1 h; 150 °C, 2 h; 250 °C, 2 h; 350 °C, 2–2.5 h; continuing until complete decolourisation to an acceptable slight yellowish tint. After mineralization was complete, the sample was alkali-treated with 30% NaOH, and the released ammonia was steam-distilled and quantitatively collected in 25 mL of 4% boric acid solution with the addition of 3.5 µL of Groak indicator (a 1:1 mixture of 0.4% methyl red and 0.2% methylene blue in pure ethanol). Finally, the distilled sample was titrated with a 0.05 M H_2_SO_4_ solution. The obtained titre value was inserted into the formula for nitrogen content determination:

$$X=\;\frac{\left(V_{1}-\;V_{0}\right)*K*0.0014*10,000}{m*(100-W)}$$

where *V*_1_ is the volume in mL of 0.05 M sulfuric acid solution used to titrate the ammonia in the sample solution; *V*_0_ is the volume in mL of 0.05 M sulfuric acid solution used to titrate the ammonia used as the ‘blank’ solution; *K* is a correction factor for the titre of 0.05 M sulfuric acid solution; 0.0014 is the amount in g of nitrogen equivalent to 1 mL of 0.05 M sulfuric acid solution; *m* is mass in g of the sample; and *W* is grain moisture content in percetage.

The protein content was calculated by multiplying the total nitrogen content by a conversion factor of 5.7, in accordance with GOST 10846-91 [33].

Gluten content (GC) and gluten index (GI) were measured with a Glutomatic-2200 system (Perten Instruments, Hagersten, Sweden), following the manufacturer’s instructions. For the analysis, a 10 g sample of whole-ground seeds was placed in the washing chamber of a Glutomatic-2200 instrument with an 88 µm polyester sieve inserted. Then, 4.8 mL of 1% NaCl solution was added to the sample and mixed for 20 s to form dough. After mixing, the washing process began automatically for 5 min, and the resulting mass of wet gluten was transferred to a special sieve cassette for 30 s, followed by centrifugation at 6000 rpm for 1 min using a Gluten Index 2015 centrifuge (Perten Instruments, Hagersten, Sweden). The gluten fraction that passed through the sieve was separated and weighed. The gluten remaining on the sieve was collected and combined with the fraction that had passed through to determine the total wet gluten weight. The gluten index (GI) was calculated as the percentage of the gluten remaining on the sieve after centrifugation relative to the total wet gluten weight.

### 2.6. Glutenin Extraction

Glutenin extraction was performed based on a method described earlier [34] with several modifications. Five crushed seeds were placed in a 2.0 mL microtube, 1 mL of 70% ethanol was added, and the samples were incubated at 65 °C for 30 min. The tubes were then centrifuged at 12,000× *g* for 5 min, the supernatant was discarded, and the ethanol extraction step was repeated once again in the same conditions. The pellet was then washed with 500 µL of 50% 1-propanol followed by centrifugation under the same conditions. The pellet was dried in a fume-hood to remove residual 1-propanol and the dry pellet then dissolved in 200 µL of extraction buffer as described in [34].

### 2.7. Glutenin Allele Classification

The separation of high-molecular weight (HMW-GS) and low-molecular weight (LMW-GS) glutenin subunits was performed in polyacrylamide gels using denaturing SDS-PAGE [35], with minor modifications [36]. Two durum wheat cvs., Langdon and Senatore Capelli, and one bread wheat reference cv. Chinese Spring were used as standards for the identification of alleles in durum wheat genotypes including cultivars and hybrid lines, based on catalogues for glutenin alleles [37,38]. The nomenclature of glutenin loci used for durum wheat was based on the Gene Catalogue for bread wheat glutenins: *Glu-A1*, *Glu-B1*, *Glu-A3*, *Glu-B3* [39]. Alleles at each locus were designated by Latin letters as a ‘genetic formula’ in the same order of genes. For instance, the genetic formula of LMW-GS in cv. Langdon was: *Glu-A3b*, *Glu-B3b*, with the following final abbreviation: ***b***,***b***.

### 2.8. Regression Analysis for Comparison of Different Methods

To compare the rapid against the classical methods, the regression formula was used, which indicates by how many units, on average, the dependent variable *y* changes when the independent variable *x* increases by one unit [40].

$$\hat{y}=b_{0}+\left(b_{1}*x\right)$$

The value of *b*_0_ was calculated using the relationship *b*_0_ = $\bar{y}$ − (*b*_1_$\bar{x}$), where $\bar{y}$ is the mean value of the dependent variable from Method 2, and $\bar{x}$ is the mean value of the independent variable from Method 1. The coefficient of determination for the regression model was calculated using the following formula: *R*^2^ = *r*^2^, where *r* is the Pearson correlation coefficient. The formula used to calculate the regression slope coefficient was:

$$b_{1}=\frac{\sum (x_{i}-\;\bar{x})(y_{i}-\bar{y})}{\sum (x_{i}-\;\bar{x}{)}^{2}}$$

where $\bar{x}$ and $\bar{y}$ are means of values for methods 1 and 2, respectively.

The significance criterion for the regression coefficient, *b*_1_, was calculated using the formula: *t_r_* = *r/s_r_*, where *r* is the Pearson correlation coefficient, and $s_{r}=\;\sqrt{\left(1-r^{2})/(n-2\right)}$.

The standard error of the slope coefficient, *b*_1_, is calculated using the formula:

$${\mathrm{SE}}_{b_{1}}=\;s_{r}\sqrt{\frac{\sum (y-\bar{y}{)}^{2}}{\sum (x-\bar{x\;}{)}^{2}}}$$

The confidence interval for the slope coefficient is calculated using the formula: *b*_1_ ± *t*_0.05_ × *s_b_*, where *υ* = *n* − 2 and *t*_0.05_ = 2.12, based on Student’s *t*-distribution Table for 95% confidence interval.

The standard error of the free intercept *b*_0_ was calculated using the formula:

$${\mathrm{SE}}_{b_{0}}=s\;\sqrt{\frac{1}{n}+\;\frac{{\bar{x}}^{2}}{\sum (x-\bar{x}{)}^{2}}}$$

where *s* value was calculated as follows:

$$s=\;\sqrt{\frac{\sum (y-\hat{y_{i}}{)}^{2}}{n-2}}$$

The 95% confidence interval (CI) for the free intercept *b*_0_ with *υ* = *n* − 2 and *t*_0.05_ *=* 2.12 was given by the formula: ${\mathrm{CI}}_{b_{0}}=\;b_{0}\pm t_{0.05}\times \;{\mathrm{SE}}_{b_{0}}$

### 2.9. DNA Extraction, PCR, Sanger Sequencing and SNP Identification

One youngest fully-developed leaf was collected from individual one-month-old plants for each DNA sample extraction, for 10 plants from each of eight biological replicates indicated in Section 2.1 above for each genotype. Leaf samples were placed in 10-mL tubes and frozen in liquid nitrogen. DNA was extracted using the CTAB method as described earlier [41] with minor modifications. Frozen leaf samples were ground with ball bearings and a Vortex-mixer, keeping samples frozen. The washed and dried DNA pellet was finally dissolved in 100 µL of 1/10 diluted TE Buffer with 25 µg of RNase added. The DNA concentration was measured by a NanoDrop spectrophotometer (Thermo Fisher Scientific, Waltham, MA, USA), and the DNA quality was assessed on a 1% agarose gel.

In the current study, two genes were used, *TdPsy-B1*, encoding phytoene synthase for carotenoid synthesis, and *TdGlu-B1*, encoding high-molecular-weight glutenin subunits (HMW-GS). To identify the correct positions of the studied genes, annotated sequences were compared.

For the *TdPsy-B1* gene, the annotated sequence EU096093 [42] was compared with TrturSVE7B02G02169600 in durum wheat, cv. Svevo, v.2, using the GrainGenes database [43]. For the *TdGlu-B1* gene, the annotated sequence EU157184 [44] was compared with TrturSVE1B02G00234380 in durum wheat, cv. Svevo, v.2, using the same GrainGenes database. As a result, primers for sequencing both genes were designed based on the reference genome of durum wheat, cv. Svevo, targeting published earlier SNPs [45], and their sequences are present in Supplementary Materials S1.

Fragments of genetic regions with 1105 bp in an intron region of *TdPsy-B1* and 1154 bp in the promoter region of *TdGlu-B1* were amplified using PCR with conditions described earlier [46], with some modifications for sequencing. Briefly, the total reaction volume was 60 μL and contained 6 μL of template DNA (20 ng/mL) with the following components in their final concentrations for each reaction: 1 × SE PCR buffer including 1.5 mM MgCl_2_, 0.2 mM of each dNTPs, 0.25 mM of each primer, and 1.0 U of E332 Taq DNA polymerase (SibEnzyme, Novosibirsk, Russia). PCR was conducted on a SimpliAmp Cycler (Thermo Fisher Scientific, Waltham, MA, USA) using a program with the following steps: initial denaturation, 94 °C for 2 min; 35 cycles of 94 °C for 15 s, 55 °C for 15 s, and 72 °C for 1 min; and a final extension of 72 °C for 3 min.

The PCR products were purified using a PCR Purification kit (Syntol, Novosibirsk, Russia) following the manufacturer’s instructions, and their concentrations were measured using NanoDrop (Thermo Fisher Scientific, Waltham, MA, USA). Sanger sequencing was carried out using BigDye Terminator v3.1 reagents and SeqStudio, Applied Biosystems (Thermo Fisher Scientific, Waltham, MA, USA) at S.Seifullin Kazakh AgroTechnical Research University, Astana (Kazakhstan). Targeting SNPs were identified in both genes, *TdPsy-B1* and *TdGlu-B1*, using manual comparison of the visualised sequences using the Chromas computer software program, version 2.0. The identified SNPs were verified by sequencing of the same PCR products in both directions and compared with targeted SNPs as published earlier [45].

### 2.10. Genotyping with ASQ Molecular Markers

Two sets of ASQ markers were developed in this study for *TdGlu-B1* and *TdPsy-B1*, respectively. Allele-specific ASQ primers were developed targeting SNPs in both *TdGlu-B1* and *TdPsy-B1* genes based on re-designing KASP markers BX7OE_866_SNP and PSY_B1c_SNP, respectively [45], and their sequences are present in Supplementary Materials S2.

The ASQ method was used for plant genotyping based on the previously published protocol [47] with the following modifications. The molecular probes with a short 4-bp tag were used, and two allele-specific forward primers together with one reverse primer were designed for each SNP in both genes, *TdGlu-B1* and *TdPsy-B1*. The composition of the PCR cocktail for ASQ genotyping and amplification protocol was the same as published earlier [48].

All primers and molecular probes were obtained from DNA Synthesis (Moscow, Russia). Each ASQ genotyping reaction had a 10-μL cocktail in total and was loaded into a 96-well microplate. PCR for ASQ genotyping and allele discrimination was performed using two types of instruments, a QuantStudio-7 Real-Time PCR system (Thermo Fisher Scientific, Waltham, MA, USA) at KATRU and a CFX96 Real-Time qPCR system (BioRad, Hercules, CA, USA) at Flinders University with automatically recorded fluorescence. Amplification of FAM and HEX was checked and controlled, whereas genotyping results were determined in a post-run step with analysis of Real-Time dRn settings using the inbuilt software for the Thermo Fisher Scientific instrument and with Maestro software ver 2.3. for the BioRad instrument. Genotyping experiments were carried out in 10 individual plants for each of eight biological replicates indicated in Section 2.1 above in each parent and hybrid population with two instrumental runs.

### 2.11. Statistical Treatment

Excel 365 (Microsoft) packages were used to calculate and analyse means, standard errors and significance levels. One-way ANOVA with additional Tukey-Kramer test or Fisher’s least significant difference test were employed for comparison of parents and hybrids with their colourimetric and biochemical analyses.

For each population, the frequency of the *p_a_* allele was calculated based on the number of plants with genotypes *aa*, *ab* and *bb* using the formula:

$$p_{a}=\;\frac{2N_{\mathrm{aa}}+\;N_{\mathrm{ab}}}{2N}$$

where *N_aa_*, *N_ab_*, and *N_bb_*—are the numbers of plants with the respective genotypes and N-is the total number of plants in the population.

The frequency of the *p_a_* allele was used to assess its association with the phenotypic trait using the linear regression method: *Y* = *b*_0_ + (*b*_1_ × *p_a_*).

To assess the differences between calculated values and actual results, the mean absolute error (MAE) and root mean square error (RMSE) were calculated [49]:

$$\mathrm{MAE}=\;\frac{1}{n}\sum \left|y_{i}-\;\hat{y_{i}}\right|\;\mathrm{and}\;\mathrm{RMSE}=\sqrt{\frac{1}{n}\sum \left|y_{i}-{\hat{y}}_{i}\right|}$$

To assess the influence of growing season conditions, a two-factor analysis of variance (ANOVA) of the traits was performed, considering genotype, growing year (2024 and 2025), and ‘genotype × year’ interaction. Effects were considered statistically significant at *p* < 0.05.

Eight biological replicates, with either bulks or 10 individual plants, were used for biochemical analyses and molecular studies, respectively, in each parent and hybrid population.

## 3. Results

### 3.1. Colour Scores in Whole-Grain Flour and Carotenoid Content in Parents and Hybrids of Durum Wheat

Carotenoid content and grain colour characteristics, determined using the CIELAB system, varied among the studied durum wheat parents and hybrids. To visualize colour differences in whole-grain flour, CIELAB values were converted to the RGB (Red, Green, Blue) system (Supplementary Materials Table S1).

**Figure 2:**
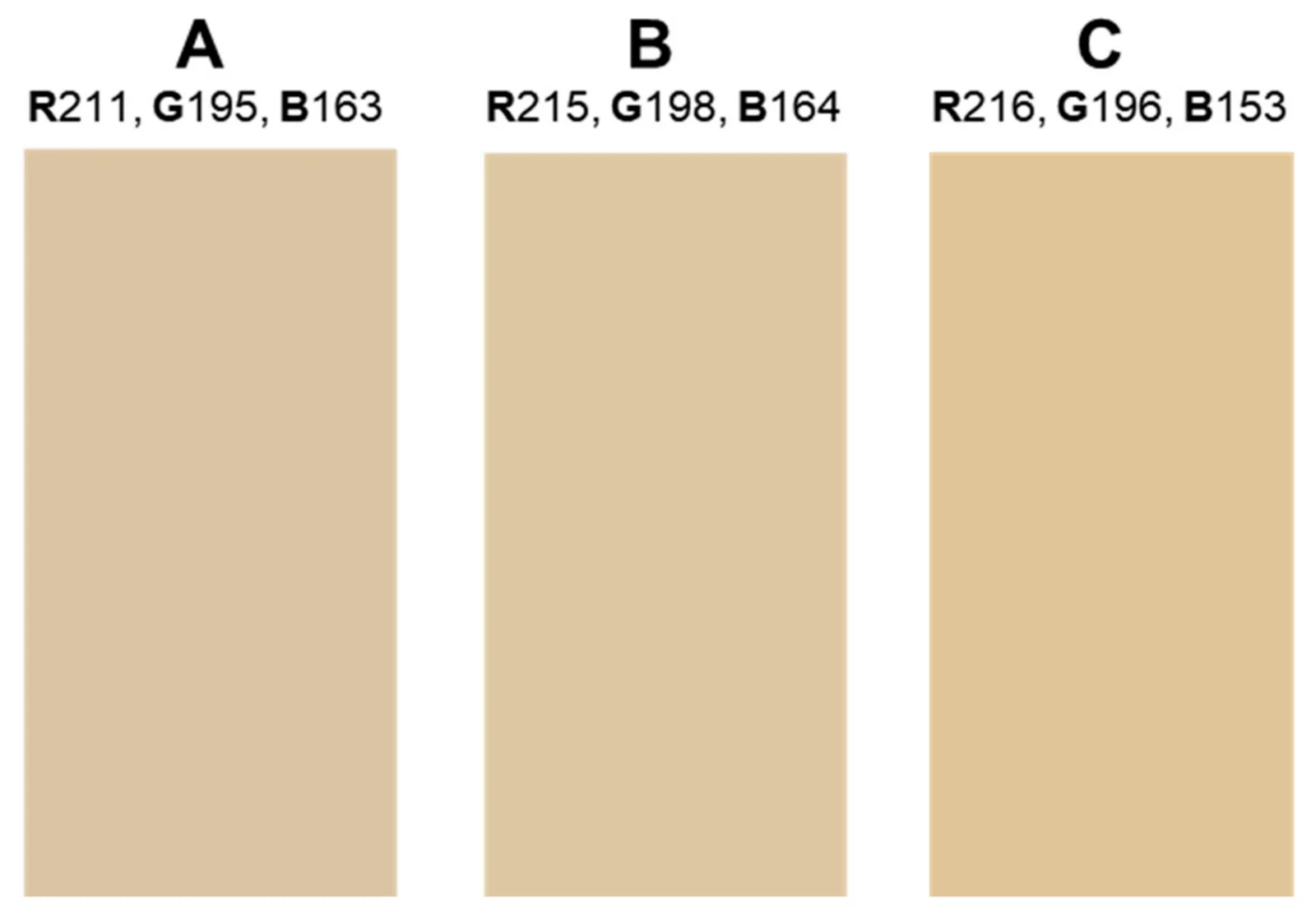
Example of colour codes of whole-ground grain in three parents. (**A**) Condure; (**B**) Hordeiforme-723; and (**C**) Levante. Parameters RGB are indicated on the top of each image.

The value of parameter L* (whiteness) ranged from units 78.55 (L × A) to 80.92 (DY × A). The range of L* values was quite narrow, indicating the similarity of the studied genotypes for grain lightness. An example of colour codes of freshly crushed grains in three parents is present in Figure 2.

The parameter a*, characterized colour shift from green to red, and ranged from unit 0.38 in Odisseo, to 0.92 in L × A. The coordinate b*, reflected colour shift from blue, −b* to yellow, +b*, varied between units 14.85 and 23.93. The highest values for this parameter were observed in cvs. Hordeiforme-723 and Levante, with units 19.57 and 23.93, respectively, as well as in the hybrids C × O (19.13), L × C (19.12), H × L (19.05), L × A (18.95), and O × H (18.93). The mean CIELAB colour coordinate values for parents and hybrids were determined to be units 79.74 for L*, 0.70 for a*, and 18.23 for b*. However, when considering parents separately, the mean values were slightly adjusted as follows: L* = 80.01, a* = 0.64, and b* = 18.22 units, whereas the following values were obtained for hybrids: L* = 79.53, a* = 0.76, and b* = 18.24 units.

Overall, the results of colour analyses indicate variability grain colour parameters among durum wheat cultivars and hybrids. The parents, cvs. Hordeiforme-723 and Levante, showed their high carotenoid content and maximum b* coordinates, whereas the hybrid combinations O × H, H × L, C × O, L × C and R × L exhibited relatively high carotenoid accumulation, which is of particular interest for further selection.

In biochemical measurements, average carotenoid content was 3.34 ppm, ranging from 1.77 (DY) to 4.81 ppm (Odisseo). Among hybrids, the highest carotenoid content was observed in H × L (3.88 ppm), O × H (3.85 ppm), R × L (3.83 ppm), and L × A (3.77 ppm). Importantly, no one hybrid studied exceeded the carotenoid content in cvs. Odisseo and Levante. However, the majority of the hybrids had significantly higher carotenoid content compared to standard cv. DY (Table 2).

### 3.2. Biochemical Analysis of Grain in Parents and Hybrids

**Figure 3:**
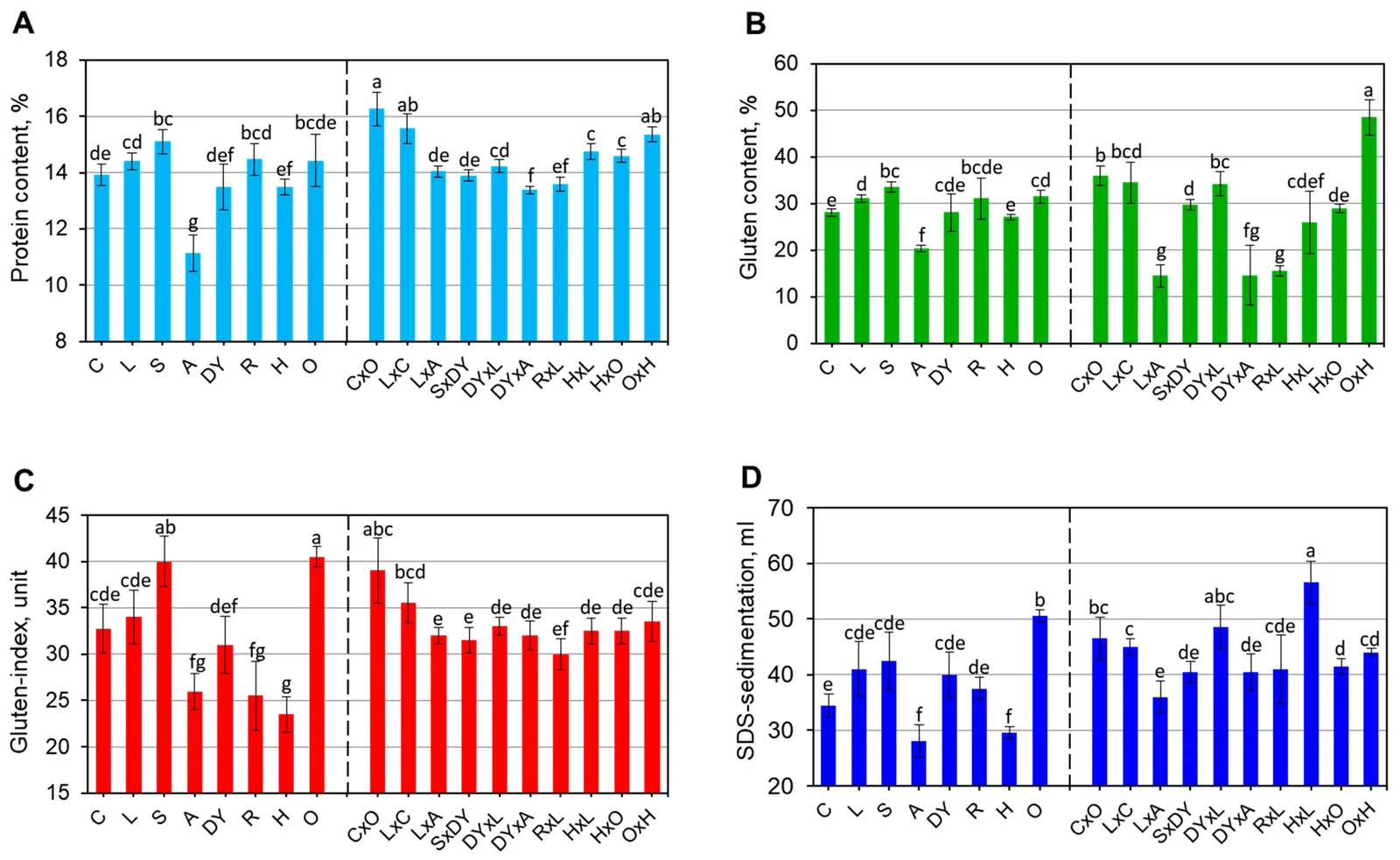
Biochemical analyses of grains in durum wheat parents and hybrids. (**A**) Protein content; (**B**) Gluten content; (**C**) Gluten Index; and (**D**) SDS-sedimentation. In each figure panel, parents are shown on the left-hand side, whereas hybrids are on the right-hand side. The bars represent the average of eight biological replicates with SE. Significant differences were calculated using one-way ANOVA (*p* < 0.05) with additional Tukey-Kramer test, shown by different letters. Shared letter indicates no significant differences between presented data. The codes of the durum wheat cultivars used are present in Table 2 as follows: C, Condure; L, Levante; S, Sycysco; A, Aktobe-1; DY, Damsinskaya Yantarnaya; R, Raya; H, Hordeiforme-723; and O, Odisseo.

The results of grain biochemical analysis in durum wheat parents and hybrids are present in Figure 3 and Supplementary Materials Table S2. The mean protein and gluten contents in the studied cultivars and hybrids were 14.3% and 30.2%, respectively. It is important to note that hybrids C × O, L × C, and O × H had a combination of elevated protein content and relatively high gluten content. The C × O hybrid exhibited the highest protein content among the genotypes studied (16.2%), which significantly outperformed both parental forms, Condure (13.9%) and Odisseo (14.4%), in protein containers. However, despite its high gluten content (36.0%), the hybrid showed no statistically significant advantage over the parents. The L × C and O × H hybrids, with high protein content (15.6% and 15.4%, respectively), significantly exceeded only one of their parents. Specifically, the protein content in L × C hybrid was significantly higher than in cv. Condure (13.9%) but did not differ from cv. Levante (14.4%). Similarly, the O × H hybrid significantly surpassed cv. Hordeiforme-723 (13.5%) in protein content but did not differ from cv. Odisseo (14.4%). The highest gluten content was observed in the O × H hybrid (48.5%). This value was significantly higher than in standard cv. Damsinskaya Yantarnaya (28.1%) and in both parents, cvs. Odisseo (31.6%) and Hordeiforme-723 (27.1%). The means of gluten index (GI) and SDS sedimentation were 32 units and 41 mL, respectively. Overall, the results of the biochemical analysis indicate significant diversity among the studied genotypes of durum wheat for grain quality traits. Among the hybrid combinations, C × O, O × H, and L × C were the most promising, as they combined high protein content with higher gluten quantity and quality in durum wheat breeding.

### 3.3. Glutenin Electrophoresis

Regarding glutenins in the hybrid populations, the HMW- and LMW-glutenin subunit patterns in parents were identified and recorded. At the *Glu-A1* locus, the null allele showed the highest frequency in both parents and hybrids, and the components were designated as 1Ax2*** and identified in only 3 of the 10 hybrids. It should be noted that neither the parental forms nor the hybrids showed the classic 1Ax2* subunit. Instead, variants 1Ax2** and 1Ax2*** were identified, which are presumably associated with the *Glu-A1* locus and are characterised by slightly higher electrophoretic mobility compared to the classic 1Ax2* subunit.

The final assignment of these components to specific *Glu-A1* allelic variants requires confirmation using allele-specific molecular markers. Two HMW-GS variants, 1Bx6 + 1By8 and 1Bx7 + 1By8, were identified at the *Glu-B1* locus. As expected, the majority of hybrid combinations were characterised by heterozygous compositions of HMW and LMW glutenin subunits, reflecting the combination of allelic variants inherited from both parents. Polymorphism in both loci, *Glu-A1* and *Glu-B1*, was observed in L × A hybrid. The S × DY hybrid had polymorphism only at the *Glu-A1* locus, whereas polymorphism in the *Glu-B1* locus was found in C × O, DY × L and H × L hybrids. Notably, the *Glu-A1* locus was in a heterozygous state in about 20% of the hybrid combinations studied (2 out of 10), whereas a 40:60 ratio among hybrids (4 out of 10) was found to be heterozygous for the *Glu-B1* locus (Table 3).

**Figure 4:**
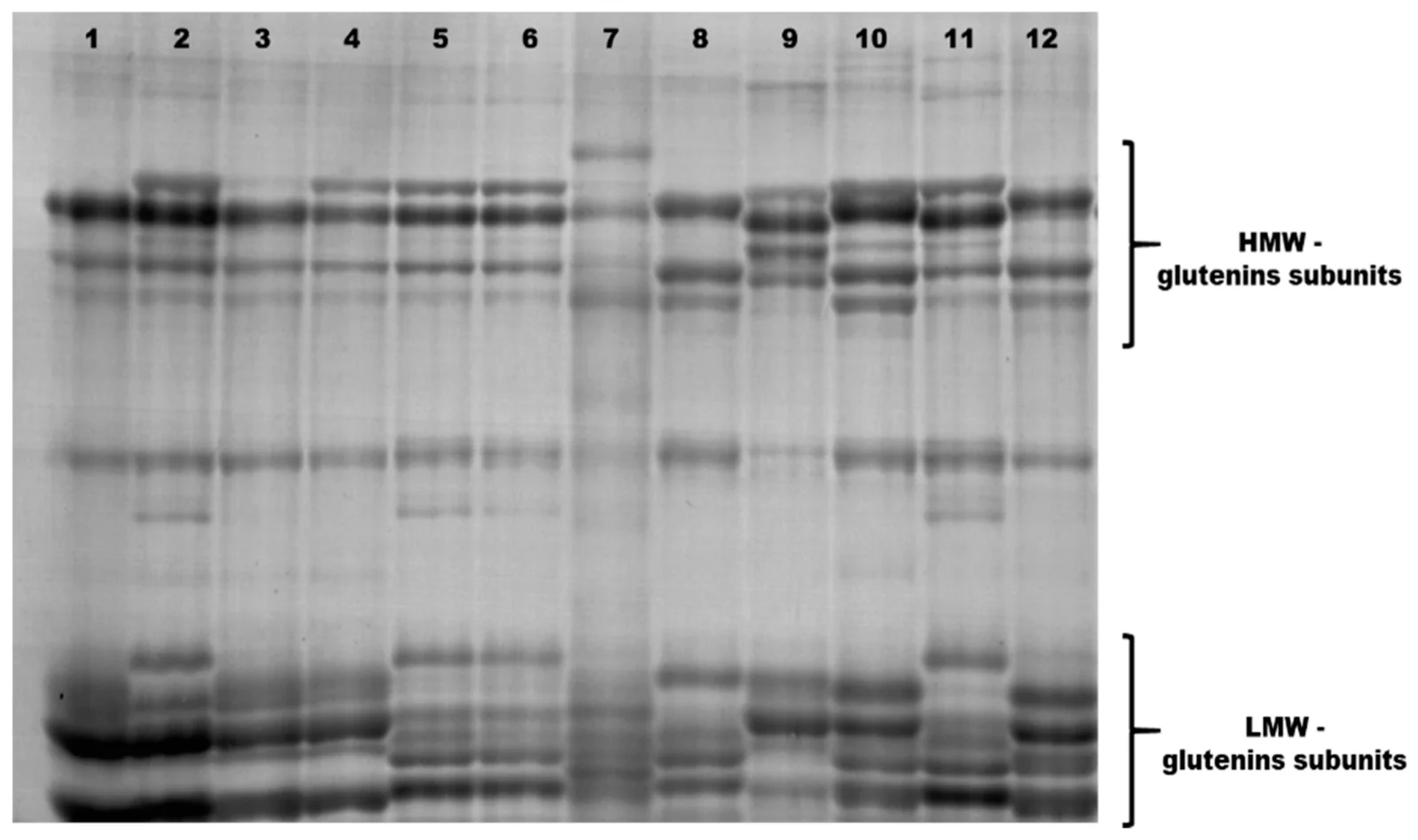
SDS-PAGE patterns of HMW- and LMW-glutenin subunits in some durum wheat hybrids and their parents. Lane 1, cv. Odysseo; 2–3, H × O; 4–5, O × H; 6, cv. Hordeiforme-723; 7, standard cv. Chinese Spring (*Triticum aestivum* L.); 8, standard cv. Langdon (*Triticum durum* Desf.); 9, standard cv. Senatore Capelli (*Triticum durum* Desf.); 10–11, H × L; 12, cv. Levante.

The electrophoretic spectrum of LMW-GS also showed limited diversity (Figure 4). Heterozygosity at the *Glu-B3* locus was detected in only two hybrids, DY × A and O × H. Based on the diagram of LMW glutenin allelic variants [37], three alleles were identified at the *Glu-A3* and *Glu-B3* locus.

It should also be noted that subunits of HMW and LMW glutenins, identified in the hybrids, generally correspond to the variants found in their parental forms. In some instances, however, the hybrids exhibited subunit combinations not observed in the parents. At the same time, the corresponding glutenin profiles remained similar to those in parents, and the observed differences appeared as combinations of parental allelic variants, the presence or absence of specific subunits, but not the occurrence of totally new glutenin variants.

### 3.4. Genotyping with KASP-Based ASQ Molecular Markers

**Figure 5:**
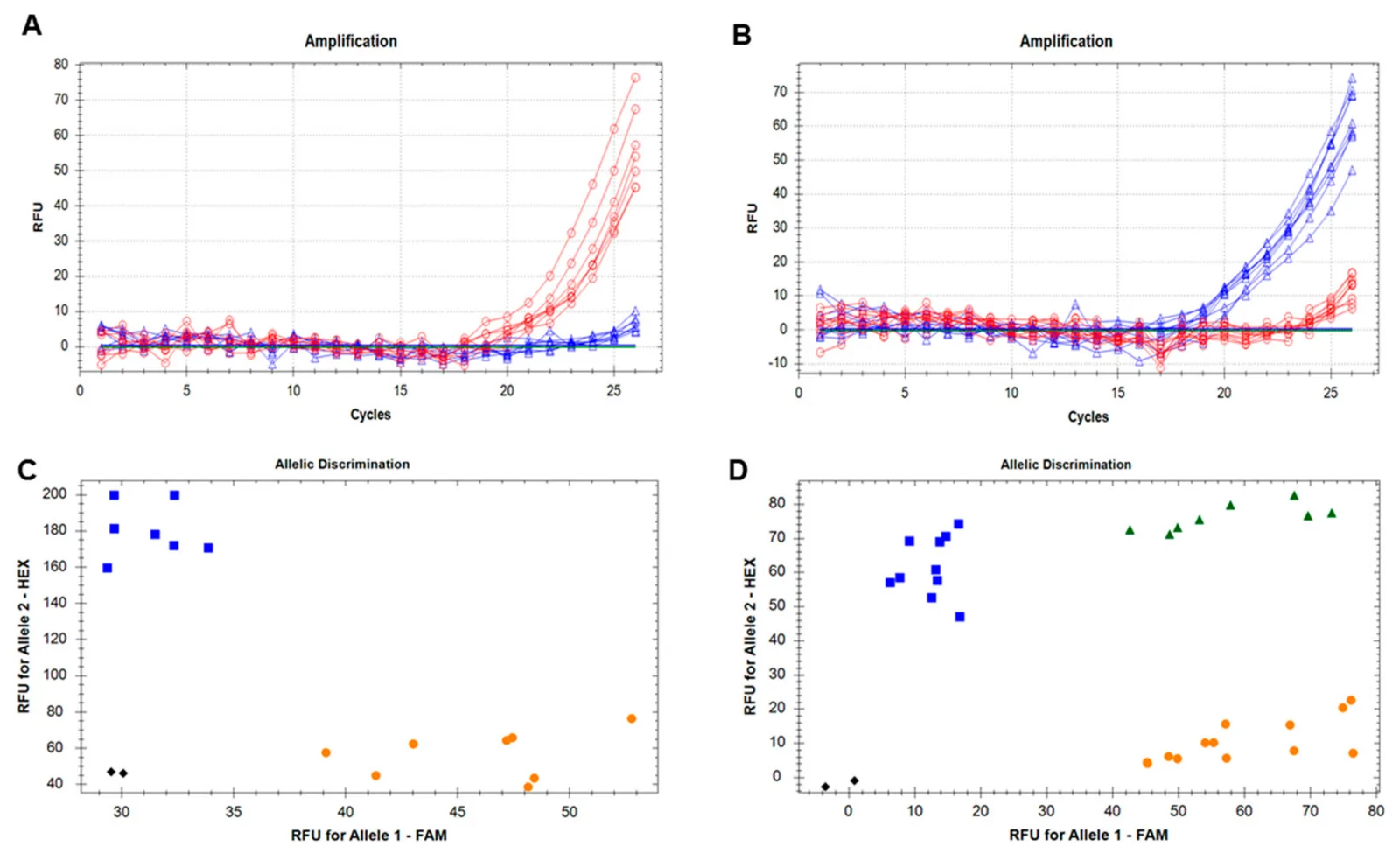
The dynamics of fluorophore amplification of FAM (**A**) and HEX (**B**) are shown in RFU (relative fluorescence units) for each cycle using red cycles for FAM and blue triangles for HEX using an ASQ marker for gene *TdGlu-B1*. Genotyping results and allele discrimination plots for parents in *TdPsy-B1* using molecular marker PSY-B1c-SNP (**C**); and for hybrids in *TdGlu-B1* using molecular marker BX7OE-866-SNP (**D**), with RFU for FAM and HEX signals, assigned in X- and Y-axes. Orange dots and blue squares represent homozygote genotypes *aa* and *bb* for alleles indicated by FAM and HEX, respectively, whereas green triangles represent heterozygotes *ab*. The allele discrimination was based on dRn automatic SNP calling. The black rhombuses show No template control (NTC) using sterile water instead of template DNA.

Sequencing results confirmed the identified and published earlier SNPs and ASQ molecular markers were developed for both genes (Supplementary Materials S1 and S2). ASQ markers TdPsyB1-PSY-B1c-SNP and TdGluB1-BX7OE-866-SNP were used for genotyping parents and hybrids for phytoene synthase gene *TdPsy-B1* and glutenin gene *TdGlu-B1*, respectively (Figure 5). The presented molecular results showed significant differences in fluorophore amplification and corresponding clear allele discriminations in both genes among studied genotypes.

To compare genotyping results with carotenoids and glutens measured by different methods, allele frequencies were already available as the primary population-level variable in hybrids with F_3_-F_4_ progenies. Therefore, allele frequencies were used as preferable points in the regression analyses based on molecular markers.

In studied 18 populations, confidence intervals of MTAs varied quantitatively between *p* = 0.012 and *p* = 0.038. Based on the calculated allele frequencies, statistically significant population-level associations between the allele *a* frequency and phenotypic traits were identified. For *TdPsy-B1*, the fitted models were b* = 15.965 + 3.312 × *p_a_* and CC = 2.351 + 1.440 × *p_a_*, with slope SEs of 0.874 and 0.368 and 95% CIs of 1.461–5.165 and 0.660–2.220, respectively. For *TdGlu-B1*, the models were SDS = 35.901 + 9.777 × *p_a_* and GI = 26.904 + 10.103 × *p_a_*, with slope SEs of 3.679 and 1.515 and 95% CIs of 1.976–17.578 and 6.891–13.316, respectively. The corresponding intercept SEs were 0.687 and 0.289 for *TdPsy-B1* and 2.533 and 1.043 for *TdGlu-B1*. The frequency of allele *a* was statistically associated with 47.3% of colourimetric b* variation; 48.9% of carotenoid content; 29.3% of the SDS-sedimentation; and 72.3% of the gluten index. These values reflect the proportion of phenotypic variation associated with the allele *a* frequency. Phenotypic data and influence of environmental factors were averaged over two years and used for the analysis (Table 4).

Presented results showed marker-trait associations (MTA) between allele frequencies, *a* and *b*, at both *TdPsy-B1* and *TdGlu-B1* loci and corresponding studied traits, carotenoid content and gluten quality. These MTA were consistent with the corresponding results obtained using rapid screening methods and traditional biochemical analyses. Therefore, the presented genotyping results with ASQ markers showed association of MTA with ASQ markers and they were consistent with the content of carotenoids and gluten quality in parents and hybrids in both methods used.

### 3.5. Correlation Analysis Between Biochemical and Colourimetric Measurements

Two-way analysis of variance showed that the year of plant growth had a significant influence on protein, gluten and carotenoid contents, as well as the sedimentation value. Protein and carotenoid contents were higher in 2024, whereas higher gluten content and sedimentation values were observed in 2025. No significant differences were found between growing years and gluten index or colour parameters (L*a*b*). Additionally, a significant genotype × environment interaction was identified for the majority of studied traits, including carotenoid content and the b* parameter, indicating that genotypes responded differently to growing conditions across different years.

The results of the correlation analysis between indicators for grain quality are presented in Table 5. Significant correlations were found between the following parameters: protein and each of gluten content, gluten index and SDS-sedimentation (*r* = 0.61–0.63; *p* < 0.05; *n* = 18); and gluten index and SDS-sedimentation (*r* = 0.66; *p* < 0.01; *n* = 18). Very high significant positive correlation was found between the colour parameter b* value and the carotenoid content (*r* = 0.76; *p* < 0.001; *n* = 18).

Linear regression analysis was performed to quantitatively characterize the relationship between the b* value and the carotenoid content. CC was used as the dependent variable, and the b* value as the independent variable. The resulting linear regression equation for CC-b* was: $\hat{\mathrm{CC}}$ = −2.577 + (0.324 × b*). The slope coefficient in this formula was 0.324 and the standard error of the slope was *s_b_* = 0.068. The significance test resulted in *t_r_* = 4.75, which significantly higher the *t*-value at the 95% significance level (Table 6).

The confidence interval for the slope coefficient at the 95% significance level was as follows: 0.324 ± 0.14 (from 0.184 to 0.464). The standard error of free intercept was SE = 1.275, and the confidence interval was CI = −2.577 ± 2.703 (from −0.125 to −5.281).

According to this equation, every one-unit increase in the b* value, the expected (calculated) carotenoid content increases by an average of 0.3244 ppm. The coefficient of determination was *R*^2^ = 0.576, indicating that the b* value explains approximately 57.6% of the variation in carotenoid content in the studied grain samples.

Thus, despite the statistically significant correlation, the b* value was characterised more by an empirical relationship rather than by the ability to quantitatively predict carotenoid content with high precision. The remaining 42.4% of carotenoid content variation was attributable to factors not accounted for by this linear model, as was confirmed by the Mean Absolute Error (MAE) and Root Mean Square Error (RMSE), with values 0.423 and 0.517 ppm, respectively. The b* value is proposed as a rapid indicator for the preliminary assessment of breeding material, rather than as a standalone method for determining carotenoid content. In other words, direct biochemical methods for carotenoid determination are required additionally for a definitive quantitative assessment.

The coefficient of correlation between GI values and the sedimentation test results was 0.66 (*p* < 0.01; *n* = 18). The coefficient of determination was *R*^2^ = 0.4334, indicating that the SDSS result, as prediction indicator, can explain 43.3% of GI value variation. Based on a linear regression analysis treating GI as the dependent variable and SDSS as the independent variable, the following equation was derived: $\hat{\mathrm{GI}}$ = 14.591 + (0.433 × SDSS). The slope coefficient in this formula was 0.433 and the standard error of the slope was *s_b_* = 0.123. The significance test resulted in *t_r_* = 3.530, which is also significantly higher than the *t*-value at the 95% significance level. The confidence interval for the slope coefficient at the 95% significance level was as follows: 0.433 ± 0.261 (from 0.172 to 0.694). The standard error of free intercept was SE = 5.183, and the confidence interval was very high as follows: CI = 14.591 ± 10.988 (from 25.579 to 3.603) (Table 6).

As with the b* value, the obtained coefficient of determination indicates limited predictive capability of the SDSS regarding the GI value. Consequently, the SDSS result should not be considered a replacement for the gluten index determination. The practical significance of SDS sedimentation lies in the possibility of using it for preliminary assessment of large amounts of plant breeding material and for selection of samples showing promise for subsequent detailed analysis involving GI determination.

Thus, the obtained statistical results indicate for statistically significant positive correlations between the indicators determined by different methods. At the same time, the moderate values of the coefficient of determination, *R*^2^, in both cases suggest that the corresponding regression models explain only a portion of the variation in the predicted indicators and cannot be considered as complete replacements. Even so, the produced regression models demonstrate the feasibility of using the colourimetric b* value for an approximate assessment of carotenoid content, and SDSS measurements for estimating expected GI values.

## 4. Discussion

It is well known that the quality of durum wheat depends on a complex set of parameters: protein content, gluten quality and quantity [50], and the content of carotenoid pigments, which provide the golden-amber colour characteristic of pasta products [5,50]. Protein content in the grain is the primary parameter influencing the subsequent quality of the final products [26,51]. This parameter prescribes the use of durum wheat with a protein content exceeding 11.5% for pasta production, whereas wheat with a protein content below 11.5% can be used as livestock feed only [52]. It should also be noted that semolina obtained from milling durum wheat is regulated based on protein content and classified into three grades: low grade (10.5–11.9% protein), medium grade (12.0–13.5% protein), and high-grade semolina (over 13.5% protein) [53]. The protein content in the hybrids studied exceeded 11.5%, which is of potential interest for the use of this breeding material in pasta production. It should be noted that pasta made from high-protein semolina is characterised by greater strength, elasticity, and resistance to overcooking compared to pasta produced from low-protein semolina. At the same time, the use of high-protein durum wheat may be associated with a lower semolina yield during milling, as well as the production of semolina with less intense colour [54,55]. Therefore, the assessment of protein content should be supplemented by characteristics regarding the quantity and, above all, the quality of the gluten. It has been established that a gluten content of at least 28% is required to produce high-quality pasta [56]. Our results indicate that 6 of the 10 hybrids studied meet this criterion. However, it should be noted that for pasta production, gluten index, GI, is a quality trait, which is much more important than seed yield and other quantitative traits. This parameter is widely used in breeding of high-quality durum wheat genotypes [57]. Gluten is considered strong at GI > 80, regular at GI = 30–80, and weak at GI < 30 [58]. In our present study, the majority of the examined cultivars and hybrids were characterised by regular gluten. The exceptions were cultivars Aktobe-1 and Hordeiforme-723, which had a GI value below 30 units (26 and 24, respectively), indicating weak gluten. Thus, the presented results demonstrate that, even with a sufficiently high protein content, some genotypes may exhibit inadequate gluten strength. This further underscores the need to simultaneously evaluate the quantitative and qualitative traits of the protein-gluten complex.

In recent years, considerable attention has been focused on developing rapid methods for assessing grain quality, particularly for early-generation breeding material. This is due to the limited amount of grain obtained from a single plant or small breeding line. Traditional methods for determination of biochemical and technological properties in wheat often require large sample size, labor-intensive sample preparation, specialised equipment, and skilled personnel [59,60,61,62]. In contrast, spectral, hyperspectral, and colourimetric methods enable the more rapid acquisition of objective quantitative estimation of samples for the preliminary screening of breeding material [63,64,65]. It should be noted that the development of rapid methods does not necessarily entail the complete replacement of traditional analytical procedures. The use of indirect indicators, statistically correlated with the results of standard methods, remains relevant for the preliminary ranking and selection of breeding samples. This approach is particularly valuable in breeding programs where a large number of genotypes must be evaluated quickly, and conducting a full suite of costly analyses for every sample is labor-intensive [66,67].

For example, the standard method for assessing the degree of grain germination is the determination of the Hagberg falling number [68], whereas modern research focuses on the possibility of assessing early stages of germination based on the activity of enzymes associated with this process [69]. A similar approach can also be applied to the assessment of gluten quality. Traditional methods, including the determination of dough rheological properties and the use of specialised systems to assess gluten characteristics, require relatively complex sample preparation and specialised equipment. The search for such a method led to the development of sedimentation analysis, an examination of the swelling of ground wheat products in water and weak acidic solutions, which provides an accurate indication of gluten and dough quality [70]. Since it was published for the first time [71], the method has undergone significant modifications adapted for application to sorghum [72], bread wheat [73], and durum wheat [23]. However, it was reported that wheat growing conditions can significantly change the efficiency of specific modifications of the method [72] and, therefore, work on refining this method is ongoing [74,75]. In light of this, the present study evaluated the feasibility of using the SDS-sedimentation test, SDSS, as a rapid and indirect indicator of gluten quality by comparing its results with the gluten index, GI, determined using the Glutomatic-2200 system in the conditions of Northern Kazakhstan.

A significant positive correlation was found between SDSS and GI (*r* = 0.66; *p* < 0.01), indicating a linear relationship between these indicators, and the coefficient of determination was *R*^2^ = 0.433. Therefore, SDSS is indeed related to GI, but this relationship was identified as moderate and does not indicate complete equivalence between the two indices. The differences between SDSS and GI are likely attributable to different aspects of the properties of the protein-gluten complex. SDS sedimentation reflects the ability of proteins to swell and form a sediment upon exposure to SDS-Na, whereas GI directly characterises the properties of isolated wet gluten during centrifugation. Therefore, the observed correlation is biologically and technologically sound, but SDS sedimentation cannot be considered as a direct replacement of GI.

In addition to the quality of the protein-gluten complex, colour is an important trait of durum wheat grain, as it significantly influences the quality of semolina and pasta products. The yellow colour is primarily associated with the content of carotenoid pigments, among which lutein plays the key role [5]. Accordingly, colour assessment can serve as an additional tool for characterizing the quality of grain and grain-derived products. The CIELAB colourimetric system is widely used for the quantitative assessment of agricultural product colour, and in this system, colour is defined by the coordinates L*, a*, and b*, representing brightness, green-red and blue-yellow axes, respectively [76]. For grain and grain-derived products, the b* component is particularly informative, as an increase in this parameter corresponds to a more intense yellow colour [77,78]. However, it was found that the particle size from milling can alter the b* value [79]. Studies comparing the colourimetric method with other analytical methods for determining carotenoid content have been conducted repeatedly.

For instance, a strong correlation has been established between b* colour value in flour of whole-ground grain and the total pigment content determined by WSB (*r* = 0.94; *p* < 0.01) and HPLC (*r* = 0.89; *p* < 0.01) methods [80]. These results confirm the feasibility of using b* parameter as a rapid preliminary indicator of carotenoid content, but it cannot replace standard analytical methods.

In the present study, this approach was used to evaluate the potential of using the b* component as a rapid, indirect indicator of carotenoid content. A highly significant positive correlation was found between b* and carotenoid content (*r* = 0.76; *p* < 0.001). A linear regression model showed that b* explains 57.6% of the variation in carotenoid content (*R*^2^ = 0.576). Thus, our presented results are consistent with published data and confirm the potential of using colourimetry for the rapid preliminary screening of durum wheat genotypes based on carotenoid content. However, similar to SDSS, this approach should be considered an indirect measurement, whereas a standard analytical method is required for the precise quantitative determination of carotenoid content.

When applying a regression model in breeding work of durum wheat, it is essential to consider MAE and RMSE values, as they are particularly important for genotypes whose trait values lie near the established selection threshold. In such cases, genotypes of breeding interest have to be verified using standard analytical methods.

Overall, the presented results demonstrate that growing conditions in different years influenced grain quality. Specifically, protein and carotenoid contents were higher in 2024, whereas gluten content and SDSS values were higher in 2025. No significant differences between years were observed for the gluten index. A significant genotype × environment interaction indicates a different response of genotypes to growing conditions. To confirm the stability of the observed patterns and assess the feasibility of their broader application, further research is required using independent breeding material obtained across different years and growing conditions.

Significant differences between two groups, *aa* and *bb*, were confirmed by genotyping using ASQ markers at both the *TdPsy-B1* and *TdGlu-B1* loci. This significant association between corresponding rapid and traditional methods is important for carotenoid content and gluten quality. The ASQ genotyping results were consistent with these phenotypic and biochemical measurements, supporting the potential use of the evaluated rapid methods for preliminary screening. However, MTAs were evaluated within the studied durum wheat material only. An independent validation population is required to demonstrate predictive performance of the markers in future studies.

The presented genotyping results with ASQ markers consistent with the phenotypic measurements within the material examined in this study to assess rapid measurement methods. However, carotenoid accumulation and gluten quality are complex quantitative traits controlled by multiple loci and, therefore, the analysis only selected SNP markers in two genes, *TdPsy-B1* and *TdGlu-B1*, showing an association with studied traits.

For breeding purposes, the results obtained confirm the advisability of a ‘step-by-step’ screening approach. In the first stage, rapid methods are used for the initial assessment of breeding material, while biochemical and technological methods are employed to refine the observed differences in quality parameters. The use of electrophoretic analysis of glutenins provides additional information on the composition of gluten proteins, which contribute significantly to gluten quality.

Genotyping using the ASQ method makes it possible to assess the allelic diversity of the target genes, *TdPsy-B1* and *TdGlu-B1*, in order to identify genotypes of breeding value. Thus, the methods presented yield complementary rather than interchangeable information; consequently, their combined application significantly facilitates and accelerates the search for and identification of promising genotypes during the early stages of the breeding process. Additionally, it should also be noted that the identified correlations do not imply that rapid methods can replace standard biochemical methods. Their practical value lies in the rapid and preliminary screening of breeding material and the identification of genotypes possessing potentially valuable quality traits.

## 5. Conclusions

(1) The rapid methods, based on colourimetry with three coordinates L*a*b* and SDS sedimentation, showed suitable preliminary estimation of carotenoid and gluten quality, respectively, in 8 parents and 10 F_3_-F_4_ hybrids of durum wheat.

(2) The comparison between results produced by rapid and traditional methods for carotenoid and gluten showed strong associations with rapid measurements and traditional methods for carotenoid and gluten quality.

(3) Genotyping results for *TdPsy-B1* and *TdGlu1-B1* genes using SNP-based ASQ markers were associated with content of carotenoids and glutenins in parents and hybrids.

(4) Colourimetric and SDS-sedimentation methods can be effectively used as rapid preliminary screening tools for selection of durum genotypes with contrasting carotenoid-related grain colour and gluten quality at the early stages of plant development. These methods can facilitate the screening of large numbers of segregating siblings in hybrid populations at early stages of breeding, while selected promising genotypes should subsequently be validated using conventional quantitative and analytical methods.

## Figures and Tables

**Table 1:** Studied accessions of durum wheat with their origin and hybrids.

Name	Code	Origin
Parents		
Condure	C	Italy
Levante	L	Italy
Sycysco	S	Italy
Aktobe-1	A	Kazakhstan
Damsinskaya Yantarnaya	DY	Kazakhstan
Raya	R	Kazakhstan
Hordeiforme-723	H	Russia
Odisseo	O	Switzerland
Hybrids		
Condure × Odisseo	C × O	F_3_-F_4_
Levante × Condure	L × C	F_3_-F_4_
Levante × Aktobe-1	L × A	F_3_-F_4_
Sycysco × DamsinskayaYantarnaya	S × DY	F_3_-F_4_
DamsinskayaYantarnaya × Levante	DY × L	F_3_-F_4_
DamsinskayaYantarnaya × Aktobe-1	DY × A	F_3_-F_4_
Raya × Levante	R × L	F_3_-F_4_
Hordeiforme 723 × Levante	H × L	F_3_-F_4_
Hordeiforme-723 × Odisseo	H × O	F_3_-F_4_
Odisseo × Hordeiforme-723	O × H	F_3_-F_4_

**Table 2:** Colourimetric results of three parameters, L*, brightness; a*, red-green; and b*, yellow-blue; and measurement of carotenoid content in the seeds of durum wheat parents and hybrids. The data are present as the average for eight biological replicates with SE. Fisher’s least significant difference, LSD, is shown in the bottom of each column.

Name	Code	Colourimetric Method (CIELab)			Carotenoid Content, ppm
		L*	a*	b*	
Parents					
Condure	C	79.35 ± 0.63	0.77 ± 0.18	18.10 ± 0.28	2.72 ± 0.29
Levante	L	79.79 ± 0.62	0.52 ± 0.19	23.93 ± 0.75	4.74 ± 0.53
Sycysco	S	80.58 ± 0.17	0.71 ± 0.16	17.50 ± 0.14	3.25 ± 0.19
Aktobe-1	A	79.81 ± 0.94	0.72 ± 0.19	16.97 ± 0.35	2.16 ± 0.11
Damsin.Yant.	DY	80.00 ± 0.88	0.55 ± 0.27	14.85 ± 1.19	1.77 ± 0.57
Raya	R	79.50 ± 1.11	0.91 ± 0.28	16.25 ± 0.96	2.16 ± 0.09
Hordeifor.-723	H	80.56 ± 0.19	0.54 ± 0.17	19.57 ± 0.87	3.95 ± 0.64
Odisseo	O	80.50 ± 0.28	0.38 ± 0.12	18.63 ± 0.29	4.81 ± 0.55
Hybrids					
C × O		79.36 ± 0.45	0.67 ± 0.18	19.14 ± 0.15	3.27 ± 0.36
L × C		78.69 ± 0.11	0.83 ± 0.13	19.12 ± 0.23	3.02 ± 0.44
L × A		78.55 ± 0.14	0.92 ± 0.21	18.95 ± 0.20	3.77 ± 0.09
S × DY		80.66 ± 0.21	0.64 ± 0.17	17.34 ± 0.17	3.16 ± 0.11
DY × L		80.22 ± 0.31	0.73 ± 0.18	16.25 ± 0.14	3.28 ± 0.09
DY × A		80.92 ± 0.30	0.75 ± 0.18	16.85 ± 0.46	3.07 ± 0.08
R × L		78.79 ± 0.20	0.88 ± 0.18	18.24 ± 0.35	3.83 ± 0.08
H × L		78.78 ± 0.21	0.82 ± 0.17	19.05 ± 0.16	3.88 ± 0.14
H × O		79.93 ± 0.51	0.60 ± 0.21	18.62 ± 0.14	3.41 ± 0.54
O × H		79.38 ± 0.25	0.73 ± 0.19	18.93 ± 0.15	3.85 ± 0.36
mean		79.74	0.70	18.23	3.34
LSD_05_		1.39	0.32	1.29	0.99

**Table 3:** Biochemical analyses of glutenin subunit formulas based on SDS-PAGE electrophoresis in seeds of durum wheat parents and hybrids. Data are represented as the average of eight biological replicates and two technical repeats.

Name	Code	Glutenin Subunit Formulas			
		*Glu-A1*	*Glu-B1*	*Glu-A3*	*Glu-B3*
Parents					
Condure	C	null	6 + 8	*a*	*c*
Levante	L	null	6 + 8	*a*	*c*
Sycysco	S	null	6 + 8	*h*	*a*
Aktobe-1	A	2***	7 + 8	*a*	*b*
Damsin. Yant.	DY	2**	7 + 8	*a*	*a*
Raya	R	2**	7 + 8	*b*	*b*
Hordeifor.-723	H	null	7 + 8	*a*	*a*
Odisseo	O	null	7 + 8	*e*	*b*
Hybrids					
C × O		null	7 + 8/6 + 8	*a*	*c*
L × C		null	6 + 8	*a*	c
L × A		null/2***	6 + 8/7 + 8	*a*	*b*
S × DY		null/2**	6 + 8	*h*	*a*
DY × L		2***	7 + 8/6 + 8	*a*	*a*
DY × A		2***	7 + 8	*a*	*b/a*
R × L		null	6 + 8	*b*	*b*
H × L		null	6 + 8/7 + 8	*a*	*a*
H × O		null	7 + 8	*a*	*a*
O × H		null	7 + 8	*a*	*a/b*

Note: The designations 2** and 2*** refer to high-molecular-weight glutenin subunits (HMW-GS) that differ in electrophoretic mobility.

**Table 4:** Genotyping results for *TdPsy-B1* and *TdGlu-B1* using ASQ markers PSY-B1c-SNP and BX7OE-866-SNP, respectively, and their comparisons with rapid and traditional methods for carotenoid and gluten quality in 8 parents and 10 hybrids of durum wheat, with eight biological replicates and 10 individual plants in each replicate. One-way ANOVA (*p* < 0.05) with additional Tukey-Kramer test was applied for association between allele frequencies, *a* and *b*, in genotypes and their corresponding phenotypes. Shared letter indicates no significant differences between presented data.

Name and Code	Carotenoid Content			Gluten Quality		
	*TdPsy-B1*	Colourimetric, b*, Units	Traditional, ppm	*TdGlu-B1*	SDS-Sedimen., mL	Gluten Index, Unit
Parents						
Condure, C	*aa*	18.10 ^c^	2.72 ^c^	*aa*	34.5 ^b^	32.8 ^b^
Levante, L	*aa*	23.93 ^e^	4.74 ^e^	*aa*	41.0 ^c^	34.0 ^b^
Sycysco, S	*aa*	17.50 ^c^	3.25 ^c^	*aa*	42.5 ^c^	40.0 ^c^
Aktobe-1, A	*bb*	16.97 ^b^	2.16 ^b^	*bb*	28.0 ^a^	26.0 ^a^
Damsin.Yant., DY	*bb*	14.85 ^a^	1.77 ^a^	*bb*	40.0 ^c^	31.0 ^b^
Raya, R	*bb*	16.25 ^b^	2.16 ^b^	*bb*	37.5 ^b^	25.5 ^a^
Hordeifor.-723, H	*aa*	19.57 ^d^	3.95 ^d^	*bb*	29.5 ^a^	23.5 ^a^
Odisseo, O	*aa*	18.63 ^d^	4.81 ^e^	*aa*	50.5 ^d^	40.5 ^c^
Hybrids						
C × O	*aa*	19.14 ^d^	3.27 ^a^	*aa*	46.5 ^c^	39.0 ^e^
L × C	*aa*	19.12 ^d^	3.02 ^a^	*aa*	45.0 ^c^	35.5 ^d^
L × A	4*aa* 3*ab* 3*bb*	18.95 ^d^	3.77 ^b^	2*aa* 4*ab* 4*bb*	36.0 ^a^	32.0 ^b^
S × DY	2*aa* 4*ab* 4*bb*	17.34 ^b^	3.16 ^a^	2*aa* 5*ab* 3*bb*	40.5 ^b^	31.5 ^a^
DY × L	3*aa* 3*ab* 4*bb*	16.25 ^a^	3.28 ^a^	4*aa* 3*ab* 3*bb*	48.5 ^c^	33.0 ^c^
DY × A	2*aa* 3*ab* 5*bb*	16.85 ^a^	3.07 ^a^	2*aa* 3*ab* 5*bb*	40.5 ^b^	32.0 ^b^
R × L	4*aa* 4*ab* 2*bb*	18.24 ^c^	3.83 ^b^	3*aa* 5*ab* 2*bb*	41.0 ^b^	30.0 ^a^
H × L	*aa*	19.05 ^c^	3.88 ^b^	5*aa* 3*ab* 2*bb*	56.5 ^d^	32.5 ^b^
H × O	*aa*	18.62 ^c^	3.41 ^b^	3*aa* 2*ab* 5*bb*	41.5 ^b^	32.5 ^b^
O × H	*aa*	18.93 ^d^	3.85 ^b^	4*aa* 4*ab* 2*bb*	44.0 ^c^	33.5 ^c^

**Table 5:** Correlations between grain quality indicators and CIELAB parameters in durum wheat samples. PC, Protein content; GC, Gluten content; GI, Gluten index; SDSS, SDS-sedimentation; and CC, Carotenoid content. CIELAB parameters: L*, Brightness; a*, Red-green; and b*, Yellow-blue indicators. Correlations indicated by asterisks were statisticaly significant (* *p* < 0.05; ** *p* < 0.01; and *** *p* < 0.001).

	PC	GC	GI	SDSS	CC	L*	a*	b*
PC	1							
GC	0.63 *	1						
GI	0.61 *	0.39	1					
SDSS	0.62 *	0.35	0.66 **	1				
CC	0.28	0.10	0.38	0.39	1			
L*	−0.26	0.07	0.01	−0.14	−0.02	1		
a*	0.07	−0.29	−0.26	−0.07	−0.34	−0.63 *	1	
b*	0.28	0.14	0.22	0.09	0.76 ***	−0.24	−0.23	1

**Table 6:** Actual and calculated values of carotenoid content and gluten index based on regression models. Mean absolute error, MAE, and root mean square error, RMSE, are shown at the bottom of each column.

Name	A. CC vs. b*			B. GI vs. SDSS		
	CC, Actual Results, ppm	CC, Calculated by Regression Formula, ppm	Rest of Regression, ppm	GI, Actual Results, Units	GI, Calculated by Regression Formula, Units	Rest of Regression, Units
Parents						
Condure	2.72	3.29	−0.57	33	30	3.22
Levante	4.74	5.19	−0.45	34	32	1.66
Sycysco	3.25	3.10	0.15	40	33	7.01
Aktobe-1	2.16	2.93	−0.77	26	27	−0.72
Damsin.Yant.	1.77	2.24	−0.47	31	32	−0.91
Raya	2.16	2.69	−0.53	26	31	−5.33
Hordeifor.-723	3.95	3.77	0.18	24	27	−3.86
Odisseo	4.81	3.47	1.34	41	36	4.04
Hybrids						
C × O	3.27	3.63	−0.36	39	35	4.27
L × C	3.02	3.62	−0.60	36	34	1.42
L × A	3.77	3.57	0.20	32	30	1.82
S × DY	3.16	3.05	0.11	32	32	−0.63
DY × L	3.28	2.69	0.59	33	36	−2.59
DY × A	3.07	2.89	0.18	32	32	−0.13
R × L	3.83	3.34	0.49	30	32	−2.34
H × L	3.88	3.60	0.28	33	39	−6.56
H × O	3.41	3.46	−0.05	33	33	−0.06
O × H	3.85	3.56	0.29	34	34	−0.14
MAE			0.423			2.595
RMSE			0.517			3.353

## Data Availability

The original contributions presented in this study are included in the research paper and in Supplementary Materials. Further inquiries can be directed to the corresponding authors.

## Supplementary Materials

The following supporting information can be downloaded at: https://www.mdpi.com/article/10.3390/life16101700/s1, S1: Primers used for studying of *TdPsy-B1* and *TdGlu1-B1* genes based on reference genome of durum wheat, cv. Svevo; S2: Allele-specific ASQ primers used for genotyping *TdPsy-B1* and *TdGlu1-B1* genes in cultivars and hybrids of tetraploid wheat; Table S1: Colourimetric measurement in whole-grain flour using CIELAB system and RGB conversion in parents and hybrids of tetraploid wheat; Table S2: Biochemical analysis of grain in parents and hybrids of tetraploid wheat, harvested in 2024–2025.

## References

[1] Saini P., Kaur H., Tyagi V., Saini P., Ahmed N., Dhaliwal H.S., Sheikh I. (2023). Nutritional value and end-use quality of durum wheat. Cereal Res. Commun..

[2] Hare R. (2017). Durum wheat: Grain-quality characteristics and management of quality requirements. Cereal Grains.

[3] Malchikov P.N., Myasnikova M.G. (2023). Development, results and prospects of the spring durum wheat breeding in Russia (Post-Soviet states). Vavilov J. Genet. Breed..

[4] Marcotuli I., Colasuonno P., Hsieh Y.S., Fincher G.B., Gadaleta A. (2020). Non-starch polysaccharides in durum wheat: A review. Int. J. Mol. Sci..

[5] Colasuonno P., Marcotuli I., Blanco A., Maccaferri M., Condorelli G.E., Tuberosa R., Parada R., de Camargo A.C., Schwember A.R., Gadaleta A. (2019). Carotenoid pigment content in durum wheat (*Triticum turgidum* L. var *durum*): An overview of quantitative trait loci and candidate genes. Front. Plant Sci..

[6] Sun T., Rao S., Zhou X., Li L. (2022). Plant carotenoids: Recent advances and future perspectives. Mol. Hortic..

[7] Lachman J., Martinek P., Kotíková Z., Orsák M., Šulc M. (2017). Genetics and chemistry of pigments in wheat grain—A review. J. Cereal Sci..

[8] Colasuonno P., Lozito M.L., Marcotuli I., Nigro D., Giancaspro A., Mangini G., De Vita P., Mastrangelo A.M., Pecchioni N., Houston K. (2017). The carotenoid biosynthetic and catabolic genes in wheat and their association with yellow pigments. BMC Genom..

[9] Malchikov P.N., Myasnikova M.G. (2020). The content of yellow pigments in durum wheat (*Triticum durum* Desf.) grains: Biosynthesis, genetic control, marker selection. Vavilov J. Genet. Breed..

[10] Hung P.V., Hatcher D.W. (2011). Ultra-performance liquid chromatography (UPLC) quantification of carotenoids in durum wheat: Influence of genotype and environment in relation to the colour of yellow alkaline noodles (YAN). Food Chem..

[11] Groth S., Wittmann R., Longin C.F.H., Böhm V. (2020). Influence of variety and growing location on carotenoid and vitamin E contents of 184 different durum wheat varieties (*Triticum turgidum* ssp. *durum*) in Germany. Eur. Food Res. Technol..

[12] Requena-Ramírez M.D., Hornero-Méndez D., Rodríguez-Suárez C., Atienza S.G. (2021). Durum wheat (*Triticum durum* L.) landraces reveal potential for the improvement of grain carotenoid esterification in breeding programs. Foods.

[13] (1990). Determination of the Yellow Pigment Content of Durum Wheat Semolina and Flour.

[14] (1999). Color and Pigments Methods.

[15] Johnson M., Kumar A., Oladzad-Abbasabadi A., Salsman E., Aoun M., Manthey F.A., Elias E.M. (2019). Association mapping for 24 traits related to protein content, gluten strength, color, cooking, and milling quality using balanced and unbalanced data in durum wheat [*Triticum turgidum* L. var. *durum* (Desf).]. Front. Genet..

[16] (2019). Colorimetry—Part 4: CIE 1976 Lab Colour Space.

[17] (1990). Determination of the Sedimentation Value—SDS Test of Durum Wheat.

[18] McDermott E.E., Redman D.G. (1977). Small-scale tests of bread making quality. Flour Mill. Bak. Res. Assoc. Bull..

[19] Axford D.W.E., McDermott E.E., Redman D.G. (1978). Small-scale tests of bread-making quality. Mill. Feed Fertil..

[20] Axford D.W.E., McDermott E.E., Redman D.G. (1979). Note on the sodium dodecyl sulphate test of bread-making quality: Comparison with Pelshenke and Zeleny tests. Cereal Chem..

[21] Dexter J.E., Matsuo R.R., Kosmolak F.G., Leisle D., Marchylo B.A. (1980). The suitability of the SDS-sedimentation test for assessing gluten strength in durum wheat. Can. J. Plant Sci..

[22] Quick J.S., Donnelly B.J. (1980). A rapid for estimating durum wheat gluten quality1. Crop Sci..

[23] Dick J.W., Quick J.S. (1983). A modified screening test for rapid estimation of a gluten strength in early generation durum wheat breeding lines. Cereal Chem..

[24] Vassiltchouk N.S., Gaponov S.N., Yeremenko L.V., Parshikova T.M., Popova V.M., Tsetva N.M., Shutareva G.I. (2009). Gluten strength estimation during durum wheat breeding (*Triticum durum* Desf.). Agrar. Bull. South-East.

[25] Al-Khayri J.M., Alshegaihi R.M., Mahgoub E.I., Mansour E., Atallah O.O., Sattar M.N., Al-Mssallem M.Q., Alessa F.M., Aldaej M.I., Hassanin A.A. (2023). Association of high and low molecular weight glutenin subunits with gluten strength in tetraploid durum wheat (*Triticum turgidum* spp. *durum* L.). Plants.

[26] Sissons M.J., Ames N.P., Hare R.A., Clarke J.M. (2005). Relationship between glutenin subunit composition and gluten strength measurements in durum wheat†. J. Sci. Food Agric..

[27] Edwards N.M., Gianibelli M.C., McCaig T.N., Clarke J.M., Ames N.P., Larroque O.R., Dexter J.E. (2007). Relationships between dough strength, polymeric protein quantity and composition for diverse durum wheat genotypes. J. Cereal Sci..

[28] Kroupina A.Y., Yanovsky A.S., Korobkova V.A., Bespalova L.A., Arkhipov A.V., Bukreeva G.I., Voropaeva A.D., Kroupin P.Y., Litvinov D.Y., Mudrova A.A. (2023). Allelic variation of *Glu-A1* and *Glu-B1* genes in winter durum wheat and its effect on quality parameters. Foods.

[29] Fiedler J.D., Salsman E., Liu Y., Michalak J.M., Hegstad J.B., Chen B., Manthey F.A., Chao S., Xu S., Elias E.M. (2017). Genome-wide association and prediction of grain and semolina quality traits in durum wheat breeding populations. Plant Genome.

[30] Singh A., Reimer S., Pozniak C.J., Clarke F.R., Clarke J.M., Knox R.E., Singh A.K. (2009). Allelic variation at *Psy1-A1* and association with yellow pigment in durum wheat grain. Theor. Appl. Genet..

[31] Metakovsky E.V., Novoselskaya A.Y. (1991). Gliadin allele identification in common wheat. 1. Methodological aspects of the analysis of gliadin pattern by one-dimensional polyacrylamide-gel electrophoresis. J. Genet. Breed..

[32] ColorDesigner LAB to RGB Converter. https://colordesigner.io/convert/labtorgb.

[33] (1991). Grain and Products of Its Processing. Method for Determination of Protein.

[34] Singh N., Shepherd K., Cornish G. (1991). A simplified SDS-PAGE procedure for separating. J. Cereal Sci..

[35] Laemmli U.K. (1970). Cleavage of structural proteins during the assembly of the head of bacteriophage T4. Nature.

[36] Utebayev M., Dashkevich S., Kunanbayev K., Bome N., Sharipova B., Shavrukov Y. (2019). Genetic polymorphism of glutenin subunits with high molecular weight and their role in grain and dough qualities of spring bread wheat (*Triticum aestivum* L.) from Northern Kazakhstan. Acta Physiol. Plant..

[37] Nieto-Taladriz M.T., Ruiz M., Martinez M.C., Vazquez J.F., Carrillo J.M. (1997). Variation and classification of B low-molecular-weight glutenin subunit alleles in durum wheat. Theor. Appl. Genet..

[38] Payne P.I., Lawrence G.J. (1983). Catalogue of alleles for the complex loci *Glu-A1*, *Glu-B1* and *Glu-D1* which code for high-molecular-weight subunits of glutenin in hexaploid wheat. Cereal Res. Commun..

[39] McIntosh R.A., Yamazaki Y., Dubkovsky J., Rogers J., Morris C., Appels R., Xia X.C. Catalogue of gene symbols for wheat. Proceedings of the 12th International Wheat Genetics Symposium.

[40] Bewick V., Cheek L., Ball J. (2003). Statistics review 7: Correlation and regression. Crit. Care.

[41] Doyle J., Hewitt G.M., Johnston A.W.B., Young J.P.W. (1991). DNA protocols for plants. Molecular Techniques in Taxonomy.

[42] He X.Y., Zhang Y.L., He Z.H., Wu Y.P., Xiao Y.G., Ma C.X., Xia X.C. (2008). Characterization of phytoene synthase 1 gene (*Psy1*) located on common wheat chromosome 7A and development of a functional marker. Theor. Appl. Genet..

[43] GrainGenes A Database for *Triticeae* and *Avena*. https://graingenes.org.

[44] Ragupathy R., Naeem H.A., Reimer E., Lukow O.M., Sapirstein H.D., Cloutier S. (2008). Evolutionary origin of the segmental duplication encompassing the wheat *Glu-B1* locus encoding the overexpressed Bx7 (Bx7^OE^) high molecular weight glutenin subunit. Theor. Appl. Genet..

[45] Rasheed A., Wen W., Gao F., Zhai S., Jin H., Liu J., Guo Q., Zhang Y., Dreisigacker S., Xia X. (2016). Development and validation of KASP assays for genes underpinning key economic traits in bread wheat. Theor. Appl. Genet..

[46] Khassanova G., Kurishbayev A., Jatayev S., Zhubatkanov A., Zhumalin A., Turbekova A., Amantaev B., Lopato S., Schramm C., Jenkins C. (2019). Intracellular vesicle trafficking genes, *RabC*-GTP, are highly expressed under salinity and rapid dehydration but down-regulated by drought in leaves of chickpea (*Cicer arietinum* L.). Front. Genet..

[47] Kalendar R., Baidyussen A., Serikbay D., Zotova L., Khassanova G., Kuzbakova M., Jatayev S., Hu Y.G., Schramm C., Anderson P.A. (2022). Modified ‘Allele-specific qPCR’ method for SNP genotyping based on FRET. Front. Plant Sci..

[48] Kuzbakova M., Khassanova G., Jatayev S., Daniyeva N., Sweetman C., Jenkins C.L.D., Soole K.L., Shavrukov Y. (2025). Longer internode with same cell length: *LcSOC1-b2* gene involved in height to first pod but not flowering in lentil (*Lens culinaris* Medik.). Plants.

[49] Willmott C.J., Matsuura K. (2005). Advantages of the mean absolute error (MAE) over the root mean square error (RMSE) in assessing average model performance. Clim. Res..

[50] Troccoli A., Borrelli G.M., De Vita P., Fares C., Di Fonzo N. (2000). Mini review: Durum wheat quality: A multidisciplinary concept. J. Cereal Sci..

[51] Cubadda R.E., Carcea M., Marconi E., Trivisonno M.C. (2007). Influence of gluten proteins and drying temperature on the cooking quality of durum wheat pasta. Cereal Chem..

[52] Dimitrios A., Wanyera R., Wamalwa M. (2023). Durum wheat: Uses, quality characteristics, and applied tests. Wheat Research and Utilization.

[53] Cecchini C., Bresciani A., Menesatti P., Pagani M.A., Marti A. (2021). Assessing the rheological properties of durum wheat semolina: A review. Foods.

[54] Matsuo R.R., Dexter J.E. (1980). Relationship between some durum wheat physical characteristics and semolina milling properties. Can. J. Plant Sci..

[55] Dexter J.E., Symons S.J. (2007). Impact of durum wheat test weight, kernel size, kernel weight and protein content on semolina milling potential. Int. Mill..

[56] Vignola M.B., Bustos M.C., Vanzetti L., Andreatta A.E., Pérez G.T. (2025). Characterization of semolina and pasta obtained from hard hexaploid wheat (*Triticum aestivum* L.) developed through selection assisted by molecular markers. Foods.

[57] Clarke F.R., Clarke J.M., Ames N.A., Knox R.E., Ross R.J. (2010). Gluten index compared with SDS-sedimentation volume for early generation selection for gluten strength in durum wheat. Can. J. Plant Sci..

[58] Oikonomou N.A., Bakalis S., Rahman M.S., Krokida M.K. (2015). Gluten index for wheat products: Main variables in affecting the value and nonlinear regression model. Int. J. Food Prop..

[59] Bean S.R., Lyne R.K., Tilley K.A., Chung O.K., Lookhart G.L. (1998). A rapid method for quantitation of insoluble polymeric proteins in flour. Cereal Chem..

[60] Wrigley C.W. (1999). Potential methodologies and strategies for the rapid assessment of feed-grain quality. Aust. J. Agric. Res..

[61] Abeshu Y., Kasahun C. (2024). Fast and non-destructive multivariate test method to predict bread wheat grain major quality parameters. Int. J. Food Prop..

[62] Kelly J.M., Bohman B., Bennett D., Taylor N.L. (2025). Rapid dough making quality analysis of wheat flour using fourier transform infrared spectroscopy and chemometrics. Food Chem..

[63] Li L., Chen S., Deng M., Gao Z. (2022). Optical techniques in non-destructive detection of wheat quality: A review. Grain Oil Sci. Technol..

[64] Safdar L.B., Dugina K., Saeidan A., Yoshicawa G.V., Caporaso N., Gapare B., Umer M.J., Bhosale R.A., Searle I.R., Foulkes M.J. (2023). Reviving grain quality in wheat through non-destructive phenotyping techniques like hyperspectral imaging. Food Energy Secur..

[65] Vergara A.E., Díaz K., Carvajal R., Espinoza L., Alcalde J.A., Pérez-Donoso A.G. (2018). Exogenous applications of brassinosteroids improve color of red table grape (*Vitis vinifera* L. cv. “Redglobe”) berries. Front. Plant Sci..

[66] Kang S.P., East A.R., Trujillo F.J. (2008). Colour vision system evaluation of bicolour fruit: A case study with ‘B74’ mango. Postharvest Biol. Technol..

[67] Fagnano M., Fiorentino N., D’Egidio M.G., Quaranta F., Ritieni A., Ferracane R., Raimondi G. (2012). Durum wheat in conventional and organic farming: Yield amount and pasta quality in Southern Italy. Sci. World J..

[68] Hagberg S. (1960). A rapid method for determining alpha-amylase activity. Cereal Chem..

[69] Mangan D., Draga A., Ivory R., Cornaggia C., Blundell M., Howitt C., McCleary B., Ral J.P. (2022). A novel enzymatic method discriminating wheat pre-harvest sprouting from late maturity alpha-amylase. J. Cereal Sci..

[70] Campbell W.P., Wrigley C.W., Cressey P.J., Slack C.R. (1987). Statistical correlations between quality attributes and grain-protein composition for 71 hexaploid wheats used as breeding parents. Cereal Chem..

[71] Zeleny L. (1947). A simple sedimentation test for estimating the bread-baking and gluten qualities of wheat flour. Cereal Chem..

[72] Kibkalo I. (2022). Effectiveness of and perspectives for the sedimentation analysis method in grain quality evaluation in various cereal crops for breeding purposes. Plants.

[73] Preston K.R., Marsh P.R., Tipples K.H. (1982). An assessment of the SDS-sedimentation test for the prediction of Canadian bread wheat quality. Can. J. Plant Sci..

[74] Morris C.F., Paszczynska B., Bettge A.D., King G.E. (2007). A critical examination of the sodium dodecyl sulfate (SDS) sedimentation test for wheat meals. J. Sci. Food Agric..

[75] Seabourn B.W., Xiao Z.S., Tilley M., Herald T.J., Park S.H. (2012). A rapid, small-scale sedimentation method to predict breadmaking quality of hard winter wheat. Crop Sci..

[76] CIE (1977). CIE recommendations on uniform color spaces, color-difference equations and metric color terms. Color Res. Appl..

[77] Humphries J.M., Graham R.D., Mares D.J. (2004). Application of reflectance colour measurement to the estimation of carotene and lutein content in wheat and triticale. J. Cereal Sci..

[78] Wang K., Taylor D., Chen Y., Suchy J., Fu B.X. (2021). Effect of kernel size and its potential interaction with genotype on key quality traits of durum wheat. Foods.

[79] Hidalgo A., Fongaro L., Brandolini A. (2014). Wheat flour granulometry determines colour perception. Food Res. Int..

[80] Fratianni A., Irano M., Panfili G., Acquistucci R. (2005). Estimation of color of durum wheat: Comparison of WSB, HPLC, and reflectance colorimeter measurements. J. Agric. Food Chem..

