# Supplementary material for: Rapid Assessment of Carotenoid and Gluten Quality Traits in Durum Wheat Hybrids Confirmed by Biochemical and Molecular Marker Analyses
Source: Life (Basel). 2026 Oct 10;16(10):1700. doi: 10.3390/life16101700 (PMC13705803; doi:10.3390/life16101700)
Supplement: Supplementary file 1 [file life-16-01700-s001.zip › Supplementary materials S1&S2.pdf]

Supplementary material S1: Primers used for studying of *TdPsy-B1* and *TdGlu1-B1* genes based on reference genome of durum wheat, cv. Svevo

Durum wheat – cv. Svevo, 2.0 genome assembly
GraneGene database. https://graingenes.org

***TdPsy-B1* gene**

>Svevo.v2. 7B chromosome: 738,591,800-738,593,000
AGATCTGCGAGG**AGTACGCCAAGACCTTCTACCT**CGGTACGGTGCTCCTTCATGCATGCT
TACTCTGTTTTTCTTGAGCCATGGTGGCAGTCTGCCGCATGCCAAGCCCATGTTCTGATG
ATCATGGAGCTCACCCGTTCATGTCTGGTCGTGCATGGCAGGGACCTTGCTGATGACAGA
GGAGCGGCGGCGCGCCATATGGGCCATCTACGGTAATCAATCTGAAACCTCACCATGCCT
GGCTTGGACCCTCCATTGTTGCTCCCCTGCTGTAGTATCAGTATGTGTCACACAGTGTCA
GTTAGTTTCAGTAATGTGACTGAAAATGGAGCTAGTTTCATTTTCACTTCAGATCGTCAG
AAAGGGCATGCCCACATTTTGCATCAGTTAAATTGCTACATATTGTATTTAACAGCAACT
TGCAAGAATCTTTGACACTCCCCAAGATTATTGGCCACTTTTGTTTTCAGTTAATGGCGT
AAACTAGTTCTGGATGCGAATAATGGCAAATAGAAACATCGTGGAACTTGCATGCTATAC
ATTTATATAGATACTCCTATATAGTATAGTCAGTGAAGAATAAAGGCCTCA**C**ATAACACT
TTTTTTATATGCCATTATGTGTGGAAGAATCAAATTAGGCTTTTTGTTGGCTAAATGGCC
TCAATAGGATCAAAGTACATGAGAAAAGTTTGCAAGAACATATTCCTCACTACTTAAGGA
TGTGAACCTGAGGTTCTGTCAGTTCTAAATGAGATATACTCTAGGCATCGAACACTTTCA
GAATCTGATGTATAGCATCATTTGTGCAGTGTGGTGCAGGAGGACAGACGAGCTGGTGGA
CGGTCCCAACGCGTCGCACATCACGCCGCAGGCGCTGGACCGGTGGGAGAGGAGGCTGGA
GGACCTCTTCGCCGGGCGCCCCTACGACATGCTCGACGCCGCGCTCTCCGACACCATCAC
CAAGTTCCCCATAGATATTCAGGTACCAGCTCGCCGGTGCATAATTGTTCAGTCCACATT
GTATGATTCTGGTAGAAGAACAGAGTGGTGGTGGATATTCCCTGTCAGCATCAGATTCTC
CCTAGACCTCACAA**TCTCAGTGCAAGATGACCGGAA**AGTCCATGATTGGTCAAAATTGTT
TCGTTTGTCGGCCTTTTATTAGTCTCTGATGCTGTTATTGAGTTGTATGAACTTTTCAC

Primers for sequencing:
TdGluB1-F1seq: AGTACGCCAAGACCTTCTACCT 22 bp,.50%GC, Tm=62C

TdGluB1-R1seq: TTCCGGTCATCTTGCACTGAGA 22 bp,.50%GC, Tm=62C
(RevCom): TCTCAGTGCAAGATGACCGGAA
Amplicon size: 1,105 bp

Targeted gene in cv.Svevo, v.2:
TrturSVE7B02G02169600
Position: Chr7B: 738,590,058-738,593,970 (- strand)
Length: 3,913 bp
Description: Phytoene synthase B1 gene

***TdGlu-B1* gene**

>Svevo.v2. 1B chromosome: 569,265,100-569,266,300

AAACTTCTA**ACCATGTCGAGTAGTGGAAGGT**AGCCTGCATGGAACATGTAAATTGATAAA
CCATCTCTCCTAAAGCCCATTTCAGGTTTTGTTGCTTGTCATTTAGTTGGATTTCCAGGC
AACACAAATCCATGCTGACTCACAATATCTATCCAATGACTCACAACATCTATCTATGCT
ATGACGTGCAAAAAAGTTGGTAGAGTTTTGGACTTATGAAAACACCTTTCCGTGACAAAT
GAACTAACTGAAACCTTTGGCCAGTCATGTCTAAAATTAACATAGGATCTTAGTAAATCC
GCGTCAAATAGTTTGATTTACACCAGAATATGGATTTTGTTTACTTCCCAAATGAGTTCA
ATTAATGTAGATTTAATTGTCCATTATGCCCTCCTATGTTTAACCTCTTATGTTCTTCTA
TGATCTCCAAGTGTTTCTTCCCTGCCCCTACCTCTTTGCATGTCCATTGTCTTTCCCTGG
CCTCCATAGACGACGCACTTCTTCTCTCGTTGGCCTTATCGCTTG**G**TCACGCCATCACTA
ATATTCCACTGTAACTAGATGTACCCCACAAGTTGTTGCCGGAATATTTTACAATATATT
TAAATGAGATTTGGTTGTATGAAACATGTATATTTGGATATGAGAACTAAAACTAAAAAC
ACATATAATTTATTGTTTTTGATTATTGTATATTTGTGAAATATTTATCAAACATAAATA
GCCAAGTAAAATGGAAATTATGCTGCATGTTTGCATGCTGAGGTGAGCTTTCTTCTATGC
ATGGTTGCATATTGAGGTGGGCATTTTCATAGGTGACGTGCTTGCATGTCGGGAGAAATG
TGTTAGTGGATGCTAGTTATTTAGACTAGAAGAGTTGAGCGCGCTTTGCTGGGCAGTTGG
GGTTTGGGTAAAAAAAAACTACTCTCTCTGTTTTAAAATATAAGGTGTATTAGTTTTTTT
TTTTGAGAAGTTAAACCTTTTAAAGTTTGATCAAATTTGTAGAGAAATACATAAATATCC
ATAATATCAAGTCGATATCATTGGATTAAGCATGAAACGAATTTTCATATTTTATTTATT
TAGTATTATGGATATTGATGTTTTTGGTTGTGAACTTGGTCAAAGAAACAGATGGAGTAT
T**TGGCTTGGTGTGTGAGGAAGAT**GATGAAGTGTATTTTCTTTTTATTTATAAAAATGCAG

Primers for sequencing:

TdGluB1-F1seq: **ACCATGTCGAGTAGTGGAAGGT** 22 bp,.50%GC, Tm=62C

TdGluB1-R1seq: ATCTTCCTCACACACCAAGCCA 22 bp,.50%GC, Tm=62C
(RevCom): TGGCTTGGTGTGTGAGGAAGAT
Amplicon size: 1,154 bp

Closest genes in cv.Svevo, v.2:

TrturSVE1B02G00234390
Position: Chr1B: 569,261,933-569,264,728 (+ strand)
Length: 2,796 bp
Description: Transposable element gene

TrturSVE1B02G00234380
Position: Chr1B: 569,259,099-569,261,995 (- strand)
Length: 2,897 bp
Description: Serine/threonine-protein kinase

Supplementary material S2: Allele-specific ASQ primers used for genotyping *TdPsy-B1* and *TdGlu1-B1* genes in cultivars and hybrids of tetraploid wheat.

***TdPsy-B1* gene**

CCAAGATTATTGGCCACTTTTGTTTTCAGTTAATGGCGTAAACTAGTTCTGGATGCGAAT
AATGGCAAATAGAAACATCGTGGAACTTGCATGCTATACATTTATATAGATACTCCTATA
TAGTATAGTCAGTGAAGAATAAAGGCCTCAYATAACACTTTTTTTATATGCCATTATGTG
TGGAAGAATCAAATTAGGCTTTTTGTTGGCTAAATGGCCTCAATAGGATCAAAGTACATG
AGAAAAGTTTGCAAGAACATATTCCTCACTACTTAAGGATGTGAACCTGAGGTTCTGTCA
[Y] = [C] or [T]

TdPsyB1SNP-F1: GTGAAGAATAAAGGCCTCACA 21bp, 43%GC, Tm=57C
TdPsyB1SNP-F2: AGTGAAGAATAAAGGCCTCATA 22bp, 36%GC, Tm=56C
TdPsyB1SNP-R: CACATAATGGCATATAAAAAAAGTG 25bp, 28%GC, Tm=58C
(RevCom): CACTTTTTTTATATGCCATTATGTG
Amplicon size: 49-50 bp.

ASQ primers:
TdPsyB1SNP-F1: GTCCTTGCGAAGGCATCCGTGAAGAATAAAGGCCTCACA
TdPsyB1SNP-F2: GTCCTTGCGAAGGCCAAC AGTGAAGAATAAAGGCCTCATA
TdPsyB1SNP-R: CACATAATGGCATATAAAAAAAGTG

***TdGlu-B1* gene**

TTGTCCATTATGCCCTCCTATGTTTAACCTCTTATGTTCTTCTATGATCTCCAAGTGTTT
CTTCCCTGCCCCTACCTCTTTGCATGTCCATTGTCTTTCCCTGGCCTCCATAGACGACGC
ACTTCTTCTCTCGTTGGCCTTATCGCTTGSTCACGCCATCACTAATATTCCACTGTAACT
AGATGTACCCCACAAGTTGTTGCCGGAATATTTACAATATATTTAAATGAGATTTGGTT
GTATGAAACATGTATATTTGGATATGAGAACTAAAACTAAAAACACATATAATTTATTGT
[S] = [G] or [C]

TdGluB1SNP-F1: GAATATTAGTGATGGCGTGACC 22bp, 45%GC, Tm=60C
(RevCom): GGTCACGCCATCACTAATATTC

TdGluB1SNP-F1: GAATATTAGTGATGGCGTGAGC 22bp, 45%GC, Tm=60C
(RevCom): GCTCACGCCATCACTAATATTC

TdGluB1SNP-R: TCTCTCGTTGGCCTTATCGC 20bp, 55%GC, Tm=60C
Amplicon size: 44 bp.

ASQ primers:
TdGluB1SNP-F1: GTCCTTGCGAAGGCATCCGAATATTAGTGATGGCGTGACC
TdGluB1SNP-F2: GTCCTTGCGAAGGCCAACGAATATTAGTGATGGCGTGAGC
TdGluB1SNP-R: TCTCTCGTTGGCCTTATCGC
